# Effectiveness of Using Mental Health Mobile Apps as Digital Antidepressants for Reducing Anxiety and Depression: Protocol for a Multiple Baseline Across-Individuals Design

**DOI:** 10.2196/17159

**Published:** 2020-07-05

**Authors:** Jamie M Marshall, Debra A Dunstan, Warren Bartik

**Affiliations:** 1 School of Psychology Faculty of Medicine and Health University of New England Armidale Australia

**Keywords:** mHealth, eHealth, mobile apps, mobile phone, anxiety, depression, single-case study

## Abstract

**Background:**

The use of mental health mobile apps to treat anxiety and depression is widespread and growing. Several reviews have found that most of these apps do not have published evidence for their effectiveness, and existing research has primarily been undertaken by individuals and institutions that have an association with the app being tested. Another reason for the lack of research is that the execution of the traditional randomized controlled trial is time prohibitive in this profit-driven industry. Consequently, there have been calls for different methodologies to be considered. One such methodology is the single-case design, of which, to the best of our knowledge, no peer-reviewed published example with mental health apps for anxiety and/or depression could be located.

**Objective:**

The aim of this study is to examine the effectiveness of 5 apps (*Destressify, MoodMission, Smiling Mind, MindShift,* and *SuperBetter*) in reducing symptoms of anxiety and/or depression. These apps were selected because they are publicly available, free to download, and have published evidence of efficacy.

**Methods:**

A multiple baseline across-individuals design will be employed. A total of 50 participants will be recruited (10 for each app) who will provide baseline data for 20 days. The sequential introduction of an intervention phase will commence once baseline readings have indicated stability in the measures of participants’ mental health and will proceed for 10 weeks. Postintervention measurements will continue for a further 20 days. Participants will be required to provide daily subjective units of distress (SUDS) ratings via SMS text messages and will complete other measures at 5 different time points, including at 6-month follow-up. SUDS data will be examined via a time series analysis across the experimental phases. Individual analyses of outcome measures will be conducted to detect clinically significant changes in symptoms using the statistical approach proposed by Jacobson and Truax. Participants will rate their app on several domains at the end of the intervention.

**Results:**

Participant recruitment commenced in January 2020. The postintervention phase will be completed by June 2020. Data analysis will commence after this. A write-up for publication is expected to be completed after the follow-up phase is finalized in January 2021.

**Conclusions:**

If the apps prove to be effective as hypothesized, this will provide collateral evidence of their efficacy. It could also provide the benefits of (1) improved access to mental health services for people in rural areas, lower socioeconomic groups, and children and adolescents and (2) improved capacity to enhance face-to-face therapy through digital homework tasks that can be shared instantly with a therapist. It is also anticipated that this methodology could be used for other mental health apps to bolster the independent evidence base for this mode of treatment.

**International Registered Report Identifier (IRRID):**

PRR1-10.2196/17159

## Introduction

### Background

Mobile health apps for smartphones and tablet devices have become a lucrative business, with worldwide expenditure estimated to be over US $92 billion [1]. Apps are increasingly being used to monitor, assess, and improve mental health. There are now more than 10,000 publicly available mental health–specific apps [2]. Most of these apps lack published evidence for their effectiveness, making it difficult for clinicians and consumers to know which app is the most appropriate [3]. Currently, choices are made using reviews and ratings available in app stores [4], but these can produce unreliable results [5].

Although effective treatments for anxiety and depression exist, many people do not access these for various reasons [6]. However, with ownership of smartphones being at 70% of the global population and rising [7], mental health apps potentially offer a partial solution to limitations in service availability and acceptability.

### Previous Research

Published reviews have found that mental health apps can be effective for reducing anxiety [8] and depression [9] with an overall effect size of small to moderate [10]. Within this research, there are some notable shortcomings, including substantial heterogeneity across studies. For example, there have been differences in dosage [11,12] duration of interventions [13,14] and the absence of long-term follow-up data [15].

Another limitation of previous research is that most of it has been carried out by individuals who have developed the app, who have stood to gain financially from its sales, and/or who were otherwise associated with it [3]. For instance, a recent review of app stores found that only 1.02% of mental health apps offering therapeutic treatment for anxiety and/or depression had been evaluated using *independent* research [3]. Furthermore, in a meta-analysis of 9 studies on apps targeting anxiety [8] and in another meta-analysis of 18 studies on apps targeting depression [9], none involved independent research or replication (note that some studies were included in both meta-analyses).

A possible reason for the lack of research on apps is the time factor for large-scale experimental designs. Specifically, randomized controlled trials (RCTs) that demonstrate the efficacy of an intervention by measuring and comparing the outcomes of matched treatment and control groups are often lengthy to conduct. This is a barrier to achieving results in a time frame that is acceptable to the profit-driven app market. In the time it takes to complete an RCT, the app being studied may have been updated or disappeared from the market altogether as newer apps with enhanced features emerge in its place. Furthermore, RCTs are not necessarily the most appropriate study design for every situation, with another limitation of RCTs being lower ecological validity [16].

### Single-Case Designs

Single-case research designs address the issue of ecological validity by testing the effectiveness of an intervention for individuals (ie, performance under real-world conditions). However, single-case designs can also control for threats to internal validity and thus test for the efficacy of a treatment. Such designs go beyond a study with a sample of one participant and involve continuous and repeated measurements, random assignment, sequential introduction of the treatment, and specific data analysis and statistics [17]. Robust results in clinical psychology and behavioral science can be demonstrated when benefits are shown in 3 to 5 cases [18-20]. A single-case design is also safer than an RCT for vulnerable participants because their well-being is monitored by gathering and analyzing data more frequently during the study, and treatment can be altered if there is a clinically significant decline in status [21,22]. For mental health apps, single-case designs are a viable alternative for accelerating the evidence base [23,24].

### Objectives and Aims

The main objective of this study is to use a single-case design to examine the effectiveness of 5 mental health apps that purport to have efficacy for reducing symptoms of anxiety and/or depression.

This study seeks to answer the following research questions: (1) Do the apps in this study provide clinically significant improvements in symptoms of anxiety and/or depression? (2) What individual characteristics of participants influence the results in this regard? and (3) What individual characteristics of the apps influence the results?

The only hypothesis to be tested in this study is based on question 1:

- Hypothesis 1: the use of apps will produce improvements in mental health and well-being in line with the 3-phase model of psychotherapy outcomes [25].

The Howard et al [25] model proposed that the outcome of any psychotherapeutic intervention will involve progressive reductions in subjective distress, then symptomatology, and, finally, an increase in overall life functioning.

## Methods

### Study Design

This study is registered with the Australian and New Zealand Clinical Trials Registry (ANZCTR), which is a primary registry in the World Health Organization Registry Network (registration number: ACTRN12619001302145p).

**Figure 1:**
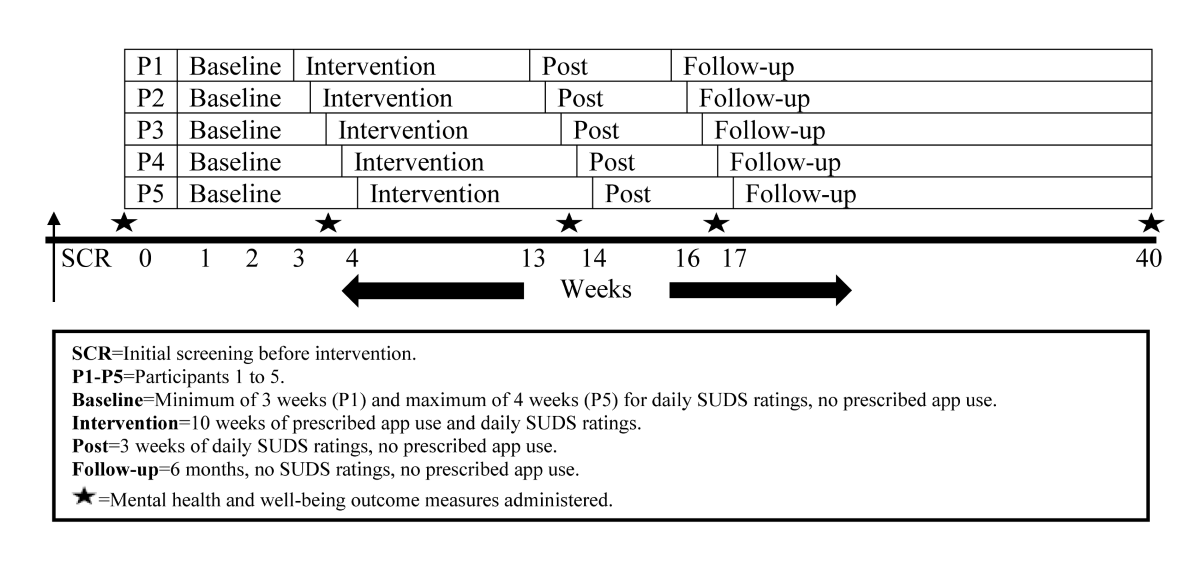
Overview of the proposed study design.

A multiple baseline across-individuals design will be employed. Multiple baseline designs in mental health intervention studies are those where a baseline period of stability of symptoms is established before the intervention is introduced. In this way, each participant acts as their own control, and internal validity is demonstrated when there is no change in symptoms until after the treatment is introduced [18]. In the design to be used in this study, all participants commence the baseline period at the same time but start the treatment at different times after a minimum number of daily data readings (at least 20) have been received. This sequential commencement approach further strengthens the internal validity by reducing the likelihood of history, maturation, or other extraneous factors explaining any observed emotional or behavioral change that occurs simultaneously with the introduction of the treatment. The multiple data recordings allow for the use of analytical techniques such as a time series analysis [20,26] and will involve participants reporting ratings of subjective units of distress (SUDS) via SMS text messaging using a 10-point scale. In this design, 4 or more baselines are recommended [18,20], and these will follow the pattern shown in Figure 1.

This research will use a *prescribed dosage* approach, as if the app was a *digital antidepressant*: one 10-min *dose* of app use per day for 5 days per week. The 10-week intervention period creates equivalence with one 50-min session per week for 10 weeks, which is the annual maximum number of psychology sessions rebated under Australia’s Medicare system [27]. The rationale for a minimum 3-week postintervention period (to demonstrate the stability of the treatment effect) is similar to that described earlier, namely, that 20 daily SUDS ratings are needed for a valid data analysis. The justification for using the 5 chosen apps are as follows: (1) each has some evidence of efficacy published in a peer-reviewed journal, (2) all have publicly available free versions, and (3) all can fit the *prescribed dosage* approach. The chosen apps use 3 popular evidence-based frameworks employed across mental health settings for treating anxiety and/or depression: cognitive behavioral therapy (CBT), mindfulness, and positive psychology. Finally, the rationale for using 10 participants per app is that, even accounting for a 50% to 60% attrition rate [28], approximately 3 to 5 participants per app will provide enough data for a valid statistical analysis using time series conventions. As previously noted, this number of replications in a single-case design with similarly presenting individuals can produce robust and generalizable findings if the results are comparable in each case [18-20].

### Recruitment

Commencing in January 2020, participants will be recruited throughout Australia by advertising the proposed study to nongovernment organizations that run programs for clients with mental illness (eg, the Benevolent Society), contacting associations of mental health professionals that may alert their members to the proposed study (eg, Australian Psychological Society), and contacting support groups and other organizations in the mental health sector (eg, Mental Health Victoria), requesting they advertise the proposed study on their various social media platforms. The advertisement is shown in Multimedia Appendix 1. Recruitment will cease once 50 participants are recruited. Owing to the nature of the proposed study design, new participants cannot commence after the study has started because the multiple baseline design requires participants to begin at the same time and then have specifically staggered phase commencements after that. Figure 1 demonstrates this process.

All 50 participants (10 for each app) will be randomized to an app and their position in the single-case design (ie, P1 to P10) using the web-based random number generator, *Research Randomizer* [29]. The full inclusion and exclusion criteria are presented in Textboxes 1 and 2, respectively. A financial reimbursement will be offered to participants of Aus $0.50 (US $0.33) per daily SUDS rating sent via a text message. The researchers acknowledge that financial payments have the potential to interfere with ecological validity, because a person in the community would not normally be paid for using a mental health app, and intrinsic motivation, because people could potentially use the app for the benefit of financial remuneration rather than for the value of improving their mental health. However, the low amount of remuneration being offered of approximately Aus $45.00 (US $29.41) on average is not considered payment for participation in the proposed study but rather reimbursement of personal expenses incurred while taking part in the proposed study. Given that the effectiveness of mental health apps has the potential to benefit those from low socioeconomic groups, being reimbursed for providing in excess of 80 text messages will alleviate reasons that a potential participant of lower socioeconomic background could provide for being out of pocket for the cost of sending text messages from their mobile phone. Therefore, it is envisaged that prospective participants will more likely have motivation to improve their mental health beyond receiving financial remuneration. In addition, this financial incentive will not be advertised, and participants will only learn about this when they provide consent when completing the demographics questionnaire.

**Study eligibility: inclusion criteria.**

Inclusion criteria:

- 18 years of age or older
- Ability to read English
- Have access to a smartphone or tablet device capable of connecting to the internet and downloading the required app and sending and receiving SMS text messages
- Agreeable to providing daily subjective units of distress ratings via SMS text messages and to completing self-report measures at 5 different time points (including 6-month follow-up)
- Mild-to-moderate anxiety and/or depression, diagnosed by a qualified health professional and confirmed by the researchers (all of whom are clinical psychologists) after screening. Screening involves analyzing the participants’ scores on the first completed set of outcome measures: the Depression Anxiety Stress Scale-21 short-form version and the Outcome Questionnaire-45 second edition version. For more information on these, see the *Mental Health and Well-Being* subsection.

**Study eligibility: exclusion and removal criteria.**

Exclusion criteria:

- Severe anxiety and/or depression, as indicated by the initial outcome measures and in any responses to specific questions in the demographics questionnaire
- History of psychosis or other complex mental health presentation as deemed by the researchers to be unsuitable for participation in this research. There will be a question in the demographics questionnaire that asks participants for their complete mental health diagnoses
- Current suicidal ideation, as indicated by a participant’s responses on the initial outcome measures

Removal criteria:

- Not providing any subjective units of distress rating for a 2-week period
- Not providing a minimum of 20 subjective units of distress ratings in the baseline and postintervention phases or a minimum of 40 subjective units of distress ratings in the intervention phase
- Not completing outcome measures either preintervention or postintervention
- Clinically significant/unsafe decline in mental health as indicated by subjective units of distress ratings or outcome measures or in the judgment of researchers
- Suicidal ideation

### Materials

Participants will supply their own smartphones and/or tablet devices. In total, 5 different apps will be used: (1) *Destressify* [30,31], (2) *MoodMission* [32-35], (3) *Smiling Mind* [36,37], (4) *MindShift* [15,38], and (5) *SuperBetter* [39,40].

All the apps are supported by published research demonstrating statistically significant efficacy for the treatment of anxiety and/or depression. Each app has an accompanying website with further information and an accessible privacy policy. Detailed information about each app and its accompanying research is provided in Multimedia Appendix 2.

### Measures

A number of measures of participants’ experiences and outcomes will be used, as described in the following sections.

#### Biographic and Demographic Features

The demographics questionnaire has been developed by the researchers to obtain information that will be examined to ascertain if any patterns in the outcome data are related to aspects of an individual’s demographic profile. Areas covered include mental health literacy [41], motivation to change [42], chronicity of anxiety and/or depression [43], and technology proficiency, all of which may influence results. Multimedia Appendix 3 contains the complete demographics questionnaire and all other measures used in this research.

#### Mental Health and Well-Being

A 3-phase model of psychotherapy outcomes [25] is applied.

1. Subjective well-being: SUDS ratings—participants rating their well-being in response to the question, “How do you feel today?,” with 0 indicating no distress and 10 indicating worst distress [44].
2. Symptoms: the Depression Anxiety Stress Scale-21 short-form version (DASS-21) [45]. Participants rate their experience of symptoms of depression, anxiety, and stress over the previous week on a 4-point scale, ranging from 0 (did not apply to me at all) to 3 (applied to me very much or most of the time). Items in each subscale are summed to provide scores for symptoms of depression and anxiety, with higher scores indicating greater severity of symptomatology. The total scores for depression and anxiety subscales are multiplied by 2 to interpret the norms [46,47]. Severity ratings for the depression subscale are 0-9 (normal), 10-13 (mild), 14-20 (moderate), 21-27 (severe), and ≥28 (extremely severe). Norms for the anxiety subscale were set as 0-7 (normal), 8-9 (mild), 10-14 (moderate), 15-19 (severe), and 20+ (extremely severe). The DASS-21 has been shown to demonstrate sound psychometric properties and validity [48].
3. Life functioning: the Outcome Questionnaire-45 second edition version (OQ-45.2) [49] is a 45-item self-report scale that measures overall interpersonal relationships and social role functioning in adults aged 18 years and older [50]. An index for overall life functioning is calculated [51]. Participants rate their feelings over the previous week on a 5-point scale, ranging from 0 (never) to 4 (always). The scale consists of both positive and negative items that are reverse-scored; higher scores indicate greater symptoms of distress and difficulties in interpersonal relations. A total score of ≥63 is indicative of clinically significant symptoms, with the subscale cutoffs for clinical significance being 35 for symptom distress, 14 for interpersonal relations, and 11 for social role [51]. The OQ-45.2 has demonstrated high internal consistency (α=.90) and test-retest reliability of *r*=.84 over a minimum 3-week period [52]. The OQ-45.2 has also shown good construct and concurrent validity in a community sample when using the total score as opposed to interpreting the 3 individual subscales [53].

#### Experience of App Usage

The Mobile Application Rating Scale-user version (uMARS) [54] is a 20-item questionnaire that records an individual’s rating on the quality of a mobile app. It contains multiple-choice and Likert-type responses and a free text field allowing users to provide a qualitative description of any aspect of the app or their experience of using the app that they wish to comment on. The uMARS contains 5 subscales: engagement, functionality, aesthetics, information quality, and a subjective quality appraisal. It has been found to have excellent internal consistency (α=.90) and good test-retest reliability [54].

### Procedure

**Figure 2:**
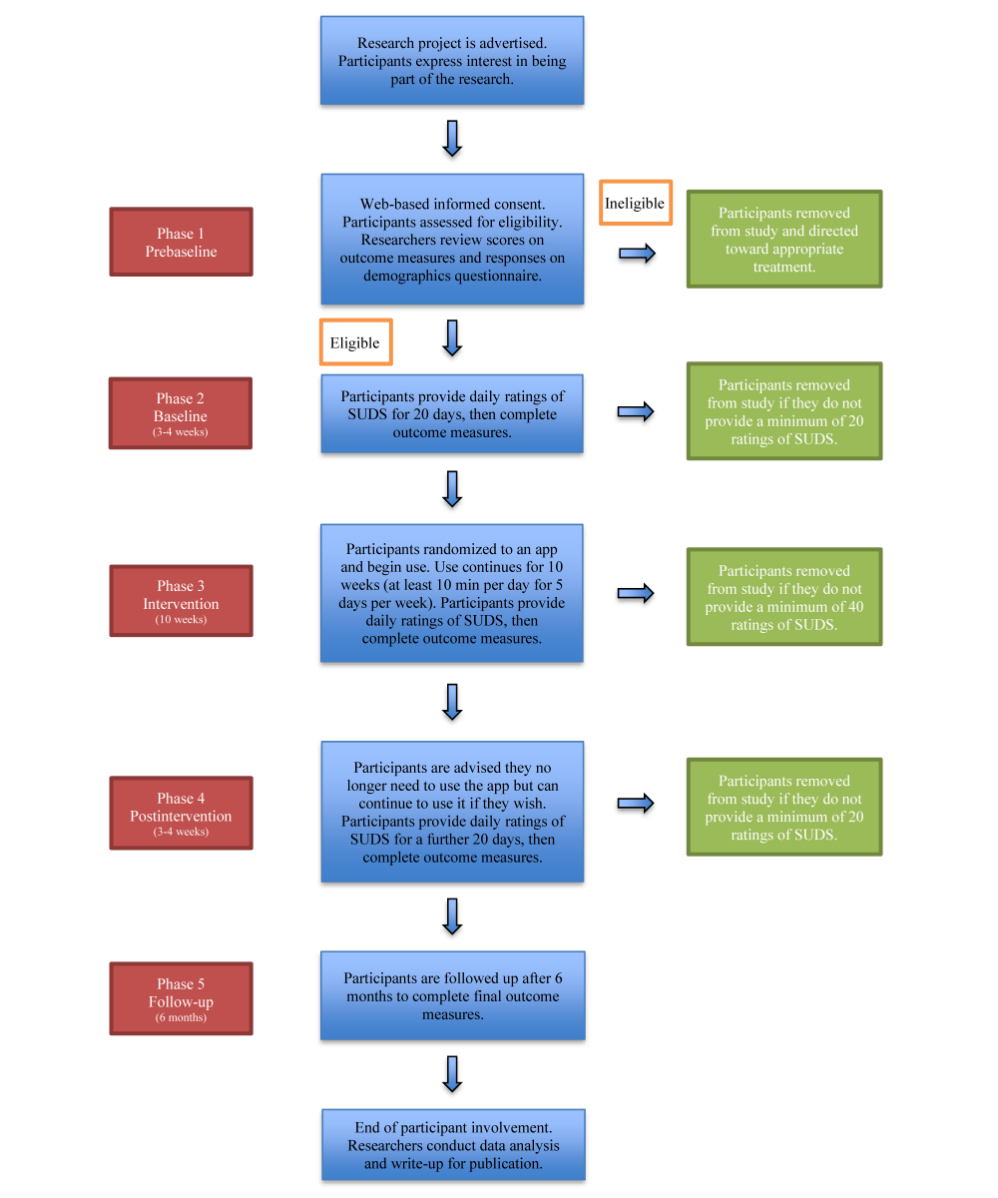
Flowchart of study and participant involvement.

Figures 1 and 2 illustrate the phases of this research. Recruitment commenced in January 2020.

#### Phase 1 (Prebaseline)

Web-based links to the information sheet for participants and consent form, the demographics questionnaire, and the mental health and well-being outcome measures (DASS-21 and OQ-45.2) are sent by email and SMS text messages and completed digitally by participants using the Qualtrics survey platform [55] and the OQ-Analyst platform [56]. Participants are screened for suitability to be in the proposed study by having their outcome measure and demographics questionnaire responses analyzed for evidence of severe anxiety and/or depression, suicidal ideation, or the presence of other severe mental illnesses such as psychosis. If researchers require further information from any participant, the participant will be contacted to clarify any queries or concerns. If a participant is deemed inappropriate for the proposed study, she/he will be directed by the researchers toward more appropriate forms of care.

#### Phase 2 (Baseline)

Accepted participants provide daily SUDS ratings for a minimum of 20 days or until a stable baseline profile of current psychological distress is achieved. At the end of this phase, the mental health and well-being outcome measures (DASS-21 and OQ-45.2) are completed.

#### Phase 3 (Intervention)

Participants are provided with generic instructions for all apps, links to both the Apple App Store and Google Play Store for their app, and specific instructions on how to use their app once it is downloaded (Multimedia Appendix 2). In addition, website links to information on the type of evidence-based framework their app uses and emergency contact information in the event of a mental health crisis are provided. Participants continue to supply daily SUDS ratings for the minimum 10-week intervention. Data analysis will be ongoing throughout this phase and will be used to assist in determining whether any participant’s mental health is significantly deteriorating. If a participant provides a SUDS rating of 10 for 2 consecutive days, they will be contacted for a check on their welfare. Similarly, if a participant’s SUDS ratings are above 8 for 5 consecutive days, they will also be contacted for a check on their welfare. We have chosen these cutoff values because the information provided to participants about the SUDS indicates that 8 is equal to their perception of feeling *very distressed* and 10 is equal to their perception of feeling the *worst distress.* The SUDS does not have a universal categorization label for each point on the scale, in addition to the number. Instead, it was designed to allow flexibility in an individual’s self-assessment [57] and labeling can vary from study to study. The mental health and well-being outcome measures (DASS-21 and OQ-45.2) and uMARS are completed at the end of this phase. If a participant’s responses on the outcome measures reveal a clinically significant decline in mental health compared with their responses at the beginning of the intervention, which places them in a severe category of mental illness, she/he will be contacted for a check on their welfare. In all cases, if a participant is categorized as being inappropriate for continuation in the proposed study, she/he will be directed toward more appropriate forms of care.

#### Phase 4 (Postintervention)

Participants provide SUDS ratings for at least 20 days following the completion of their official intervention period. Once a minimum of 20 SUDS ratings have been received, they will complete another DASS-21 and OQ-45.2. Participants are given information on all the apps so that they may explore the others if they wish.

#### Phase 5 (Follow-Up)

Participants are followed up at 6 months, where they will be asked to complete the mental health and well-being outcome measures (DASS-21 and OQ-45.2).

#### Expected Time Frames

The daily SUDS text messages will take a few seconds to reply to; app use will be a minimum of 10 min per day, 5 days per week for 10 weeks; the mental health and well-being outcome measures (DASS-21 and OQ-45.2) are expected to take less than 10 min each to complete on 5 different occasions; and the demographics questionnaire completed in phase 1 and the uMARS questionnaire completed in phase 3 are expected to take 15 min each.

The proposed study will run for approximately 40 weeks. Data analysis will be completed by approximately December 2020. A write-up for publication is expected to be completed by January 2021.

### Data Analysis

#### Descriptive Statistics and Qualitative Accounts

Descriptive statistics will be used to compare individuals and augment other analytical techniques. The data obtained from the uMARS will be used to assist in gaining an enhanced understanding of participant attitudes toward their app. Depending on the amount of qualitative information provided by participants, it will be converted via a *content analysis* [58] and will be coded into networks that hierarchically classify, identify, and summarize key themes. Data obtained from the uMARS will be plotted, as explained in the Visual Inspection subsection.

#### Time Series Analysis

A process for conducting time series analyses for psychological research was described by Borckardt et al [59] using the *R* statistical software package. In the proposed study, the commencement of the intervention will be the predictor in a regression model that uses data before and after this point to determine if there has been a statistically significant impact on subjective distress, as measured by SUDS ratings. A minimum of 20 data points are required in each phase [20,26]. The *R* statistical package will use conventions of autoregressive integrative moving average (ARIMA) modeling to account for autocorrelated data [60] when building the model.

The time series analyses [59] will evaluate statistically significant changes across the phases of the proposed study. Overall level and trends across time will be considered, and if necessary, adjustments will be made for irregular variation effects. An *irregular factor* is similar to the error terms used in many statistical models, such as generalized linear modeling. The methods of making such adjustments differ depending on the nature of the collected data but may include the augmented Dickey-Fuller test, Durbin-Watson test and/or the Ljung-Box test as part of an ARIMA model.

#### Clinical Significance and Statistical Reliability

Meaningful or clinically significant changes occur when an individual is in the dysfunctional (clinical) range at the commencement of treatment and in the functional (nonclinical) range at the end of treatment [61,62]. The clinical significance index (CSI) indicates whether individuals have made meaningful improvements to their emotional health and moved from being clinically dysfunctional to functional [63]. The reliable change index (RCI) verifies the statistical significance of any change in an individual’s score from pre- to postintervention [62]. This approach is particularly useful for single-case designs because it allows researchers to focus on individual functioning [64] and to adjust treatment if necessary. Jacobson and Truax [62] developed a classification system to describe the change in a participant’s mental health in a study’s conclusion: *recovered*=clinically significant and statistically reliable; *improved*=not clinically significant, but statistically reliable; *unchanged*=not clinically significant or statistically reliable; and *deteriorated*=clinically significant and/or statistically reliable in a worsening direction.

To determine the CSI, a cutoff point between the scores obtained by the functional and dysfunctional populations on a particular measure is identified [61,62]. Scores on either side of this point are statistically more likely to indicate whether an individual is functional or dysfunctional [61,62]. Normative data are required for both functional and dysfunctional populations for the measures being used. The CSI is based on the following formula [62]:


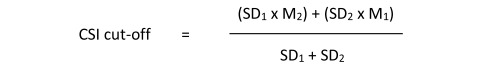


where 1 represents the nonclinical population and 2 represents the clinical population.

The RCI is a function of a measure’s standard deviation and reliability [61]. It measures an individual’s change in self-reported score from pretreatment to follow-up for statistical reliability. If an individual’s change exceeds 1.96 times the SE, the change is statistically reliable at *P*<.05 because it is unlikely to occur more than 5% of the time as a result of measure discrepancy or chance [61]. The RCI is calculated as follows [62]:


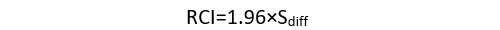


where S_diff_=√2(S_E_)^2^ and S_E_=*SD* of both groups×(√1−test-retest reliability).

Clinical significance will be calculated based on participants’ scores on the mental health and well-being outcome measures (DASS-21 and OQ-45.2) across the various phases using the framework suggested by Jacobson and Truax [62]. Using the OQ-Analyst platform, clinical significance will be compared with statistical significance and visual inspection.

#### Visual Inspection

**Figure 3:**
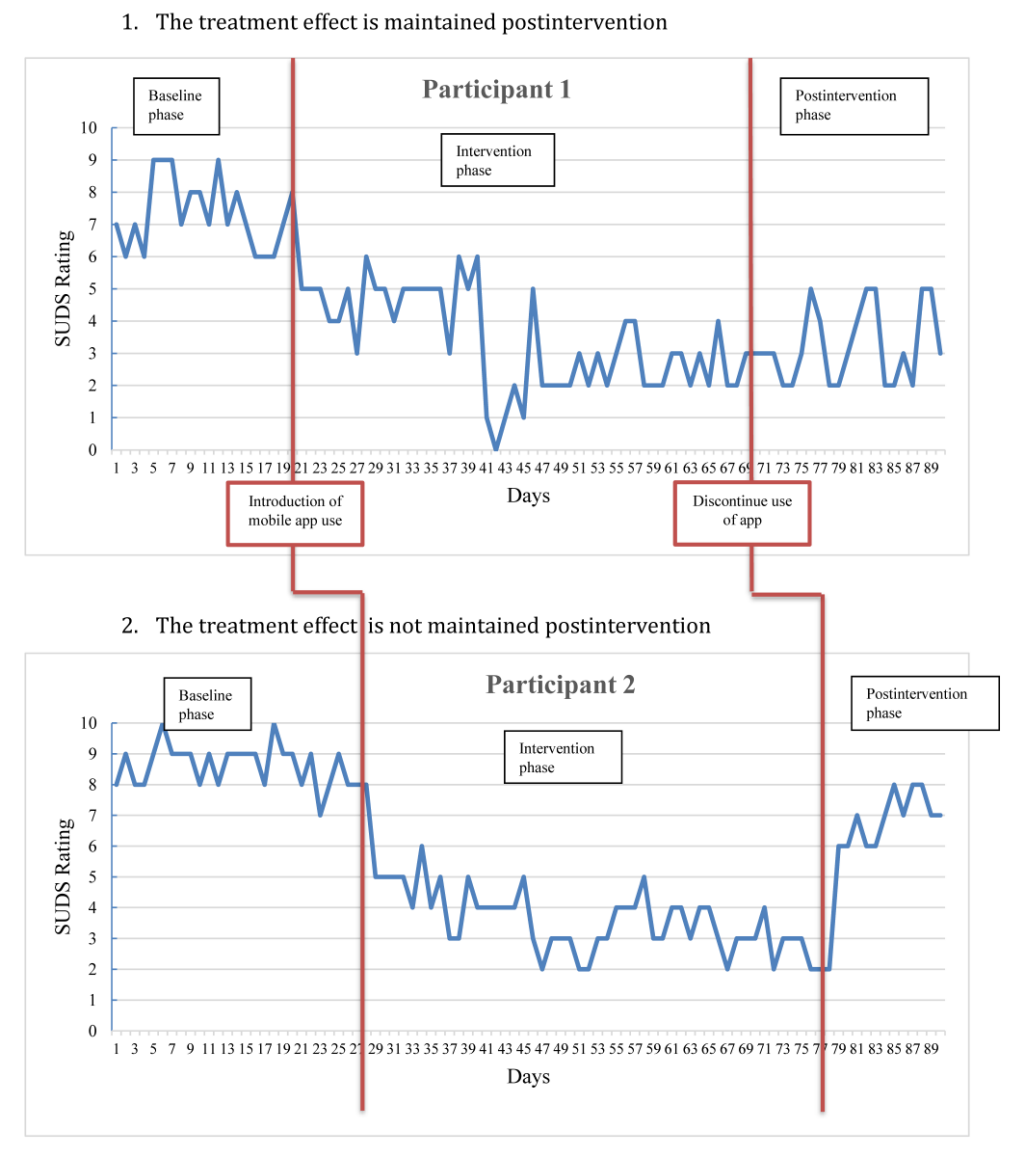
Example of how participant data may be affected and graphed using a multiple baseline across-individuals design.

Visual inspection of plotted data allows for a personal judgment about the effect of an intervention and can often produce more meaningful information than approaches involving the calculation of statistical significance [20]. In this study, visual inspection will be possible using up to 112 data points of SUDS ratings, as illustrated in Figure 3. Data obtained from the uMARS will be plotted against participant ratings from the mental health and well-being outcome measures (DASS-21 and OQ-45.2) and SUDS data and inspected for any observed relationships.

#### Data Management

Management and storage of data will occur in line with the Management and Storage of Research Data and Materials Policy of the University of New England [65]. Specifically, all nondigital materials will be scanned and digitally stored indefinitely with all other digital information pertaining to this research on the University of New England research data cloud storage facility. Digital information will be password protected and accessible only to appropriate research staff.

#### Data Exclusion

Data will be excluded from time series and visual analyses if a participant fails to provide a minimum of 20 SUDS ratings in phase 1 (baseline) and/or phase 4 (postintervention) and 40 SUDS ratings in phase 3 (intervention). Data will be excluded from CSI and RCI analyses if a participant fails to complete baseline and/or postintervention mental health and well-being outcome measures (DASS-21 and OQ-45.2). Owing to the nature of the proposed study design, participants who dropout because they did not provide the required minimum data cannot be replaced by new participants once the baseline phase has commenced.

### Ethics Approval

Ethics approval was granted by the University of New England Human Research Ethics Committee on November 1, 2019 (approval number: HE19-186). This research will be conducted under the guidelines of the National Statement on Ethical Conduct in Human Research by the Australian National Health and Research Council [66]. Any changes to procedures outlined in this protocol will be forwarded to the University of New England Human Research Ethics Committee for approval before implementation. Such changes will also be acknowledged on the trial registration at the ANZCTR.

## Results

Reporting of results will follow the Consolidated Standards of Reporting Trials of Electronic and Mobile Health Applications and Online Telehealth [67] guidelines. The Procedure section provides the estimated timelines.

## Discussion

### Principal Findings

Information about and descriptions of the previous research on each app are provided in Multimedia Appendix 2. Previous research on mental health apps is lacking, and there are other issues impacting this research. The methodologies employed in the studies based on the apps used in the proposed study are heterogeneous, and this is in keeping with other previous research on other mental health apps [3]. The studies here had varying attrition rates (the *Destressify* research [30] reported 19.9%; *MoodMission* [34], 54.8%; *Smiling Mind* [36], 17.7%; *MindShift* [15], 46.7%; and *SuperBetter* [39], 73.9%), but the varying intervention times and methodologies may have contributed to these attrition rates. Some studies were conducted by researchers who either developed the app or had an otherwise pre-existing association with the app being tested (in the case of *MoodMission* and *SuperBetter*). Other studies had participants with varying degrees of severity of anxiety and depression at commencement that were measured with varying outcome instruments (*Destressify*, *MoodMission,* and *Smiling Mind* did not specify the participants’ mental health status in their inclusion criteria; *MindShift* had participants with moderate-to-high levels of anxiety, as indicated by their ratings on at least one scale of the Patient Health Questionnaire; and *SuperBetter* had participants who scored higher than 16 on the Center for Epidemiological Studies Depression Scale). Nevertheless, all studies were published in peer-reviewed journals and authored by individuals who have associations with legitimate academic institutions and mental health organizations. Therefore, we hypothesize that our results will reveal the apps to be effective for reducing symptoms of anxiety and depression, as reflected in the previous research.

### Potential Added Value for Clinicians and Consumers

This study is unique with respect to any published study of mental health apps that could be located. This independent research, using a single-case methodology, allows for an in-depth examination of personal factors that may impact the effectiveness of these apps. It will therefore add value to existing studies on these specific apps. However, it is also anticipated that this research will be automated to the point where the design can be used to examine larger samples with the rigor of an RCT experimental design, thereby having a positive impact on increasing future research at a faster rate. It also provides an opportunity for evidence-based mental health treatment to reach those who are not already receiving it.

If mental health apps have demonstrated effectiveness, they could be incorporated by clinicians into face-to-face therapy to enhance the experience of consumers. For example, some apps allow users to complete homework tasks set by their therapist or to make thought diary entries that can be shared digitally with their therapist. Other information such as physiological readings and user-entered information such as SUDS ratings can also be sent digitally to clinicians to gain a more accurate reading of their client’s emotional health between sessions [68].

There are potential benefits for health systems and mental health consumers if apps can gain increased legitimacy for their ability to effectively manage anxiety and depression. These include the following: more economical for low socioeconomic groups to obtain mental health treatment compared with face-to-face services [69], improved access for those in rural areas where there may be limited treatment options [70], reduced stigma [71] because of anonymous assistance, access for children and adolescents who are already large consumers of smartphones and the internet [72], and it is simply a preferred way to receive mental health information for some [73]. Therefore, it is important to increase research on the efficacy and effectiveness of mental health apps using appropriate and scientifically validated methodologies in addition to RCTs, as the widely considered gold standard of RCTs may not be the most appropriate for analyzing mental health apps [23,74].

This study will examine a number of mental health apps that differ in several ways: (1) having different theoretical frameworks (*MoodMission* and *MindShift* use CBT, *Destressify* and *Smiling Mind* use mindfulness, and *SuperBetter* uses positive psychology), (2) being developed by different teams in different countries (*Destressify* was developed in the United States by individuals with an interest in mindfulness meditation, *MoodMission* was developed in Australia by a team of psychologists and researchers at Monash University, *Smiling Mind* was set up as a not-for-profit organization in Australia by mental health and meditation experts, *MindShift* was developed in Canada by a not-for-profit mental health organization, and *SuperBetter* was developed in the United States by a game designer and mental health researchers from Stanford University and the University of Pennsylvania), and (3) containing different aesthetic qualities and types of activities with different aims (*Destressify* focuses on reducing stress, *MoodMission* focuses on providing short activities designed to help an individual in response to how they are feeling at that time, *Smiling Mind* focuses on teaching mindfulness skills in a structured format using guided meditation, *MindShift* focuses on reducing anxiety by using a number of different interventions such as graded exposure and using a thought journal, and *SuperBetter* is very colorful with a playful tone that may appeal to individuals who like video games). Multimedia Appendix 2 provides further information about each app. Having a diversity of apps is important because there may be differences in the way consumers react to different aspects of an app. It is known that face-to-face therapy outcomes can be influenced by client-therapist rapport [75], client motivation [42], and chronicity/history of mental illness [43]. Therefore, there may be different aspects of a mental health app that contribute to its effectiveness, such as gamification, aesthetics, usability/interface [76], and evidence-based framework.

In sum, the reasons mentioned earlier support the need for a vigorous research agenda on the effectiveness of mental health apps, and this study methodology can assist in realizing this.

### Limitations and Strengths

This study has some limitations. First, it may not be possible to generalize the findings if the outcomes for participants with the same condition differ in significant ways. Second, there is no certainty that participants will provide daily SUDS ratings for 16 weeks, despite the minimal effort involved. Third, with many brands of smartphones using different versions of software, there is a risk that the technology between phone and app may not be compatible for some participants.

This study also has several strengths. By using questionnaires that consider subject distress, symptoms, and life functioning at different time points, the design allows for a comprehensive approach toward the impact of the apps. The use of the DASS-21 and OQ-45.2 questionnaires at multiple time points will allow an examination of issues such as suicidality, dysfunctional coping activities (such as excessive alcohol consumption), physical health, and sleep disturbance. The single-case design will also provide in-depth information about individual responses and offers a way that clinicians may be able to contribute to the evaluation process (see the Conclusions section). Finally, there is the ambitious goal of offering a future methodology that could be applied to larger RCTs via a highly digitized procedure.

### Conclusions

The evidence base for mental health apps that offer treatments for anxiety and depression is currently low. This study may assist in improving this situation in several ways. First, it may allow more clinicians to participate in the research process. Marshall et al [74] have outlined a proposal to establish a centralized database where clinicians and researchers contribute information and data on the effectiveness of mental health apps by using a standardized protocol that forms the basis of this research. Such a repository of information on mental health apps would mean an ever-increasing knowledge base that clinicians, researchers, government authorities, and academic institutions could refer to. Although there are existing websites that offer professional reviews with useful insights into mental health apps (eg, *PsyberGuide* [77], *Head To Health* [78], *Reachout Australia* [79], *Health Navigator* [80], and the *NHS App Library* [81]), these are based on professionals’ perspectives and not systematic and scientific observations. The results of increased research, such as that outlined in the proposed study, have the potential to add valuable empirical data to such websites to reinforce the reviews posted there.

If a collaborative scientific methodology was used by clinicians and researchers to rate the effectiveness of mental health apps, this would also potentially allow more transparent categorization of mental health apps in the various app stores [74]. Currently, reliance on app stores leads to potential confusion for consumers as ratings and reviews may be unreliable or even fake [5]. Using the methodology in this study is one way that, if willing, the app stores could *certify* mental health apps as having reached an acceptable level of independently verified effectiveness [3]. This would allow consumers to more clearly identify apps validated by scientific research.

Finally, given the large number of consumers who own a smartphone globally [82], if more people are able to use efficacious mental health apps on their phones, it could potentially free up scarce face-to-face services in communities struggling to meet the demand for interventions to address mild-to-moderate mental health problems.

## Appendix

Multimedia Appendix 1 Poster used to attract participants.

Multimedia Appendix 2 Information about the apps used in the present study.

Multimedia Appendix 3 Forms and measures used in the present study.

## Abbreviations

ANZCTR: Australian and New Zealand Clinical Trials Registry
ARIMA: autoregressive integrative moving average
CBT: cognitive behavioral therapy
CSI: clinical significance index
DASS-21: Depression Anxiety Stress Scale-21 short-form version
OQ-45.2: Outcome Questionnaire-45 second edition version
RCI: reliable change index
RCT: randomized controlled trial
SUDS: subjective units of distress
uMARS: user version of the Mobile Application Rating Scale
