# Supplementary material for: Effectiveness of Using Mental Health Mobile Apps as Digital Antidepressants for Reducing Anxiety and Depression: Protocol for a Multiple Baseline Across-Individuals Design
Source: JMIR Res Protoc. 2020 Jul 5;9(7):e17159. doi: 10.2196/17159 (PMC7381081; doi:10.2196/17159)
Supplement: Multimedia Appendix 1 [file resprot_v9i7e17159_app1.pdf]

Multimedia Appendix 1

Poster Used to Attract Participants

ARE YOU INTERESTED IN USING A MOBILE APP TO HELP IMPROVE YOUR MENTAL HEALTH?

Researchers from the University of New England in Armidale are looking for volunteers to use an app on their mobile phone for at least 10 weeks in an effort to help reduce the symptoms of anxiety or depression.

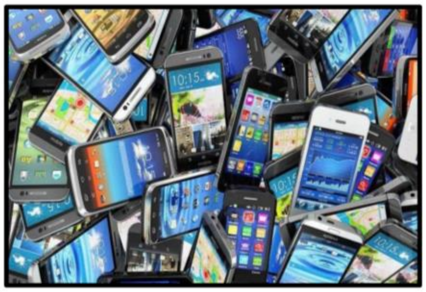

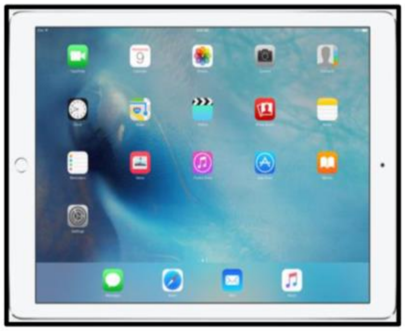

People with a previous diagnosis of anxiety or depression are encouraged to participate.

This could be a great opportunity for you to consider a new way of helping to improve your mental health!

If you would like to register, or would like more details about this project, please contact the Student Researcher, Jamie Marshall:

E-mail: jmarsh21@myune.edu.au
Phone: [redacted] during business hours

une
University of New England

This project has been approved by the Human Research Ethics Committee of the University of New England (Approval No. HE19-186, valid to 01/11/2020)
