# Supplementary material for: Effectiveness of Using Mental Health Mobile Apps as Digital Antidepressants for Reducing Anxiety and Depression: Protocol for a Multiple Baseline Across-Individuals Design
Source: JMIR Res Protoc. 2020 Jul 5;9(7):e17159. doi: 10.2196/17159 (PMC7381081; doi:10.2196/17159)
Supplement: Multimedia Appendix 2 [file resprot_v9i7e17159_app2.pdf]

Multimedia Appendix 2

Information About the Apps Used in the Present Study

Figure 4a. Participant app information sheet for *Destressify*, page 1.

APP INFORMATION

Destressify

Background

- Destressify was developed in the United States by individuals interested in mindfulness meditation.
- More information about Destressify can be found on their website: https://www.destressify.com/index.php
- Research on this app has been published: *Lee, R. A., & Jung, M. E. (2018). Evaluation of an mHealth app (Destressify) on university students' mental health: Pilot trial. JMIR Mental Health, 5(1), e2.*
- Destressify is based on a technique called mindfulness, and has a focus on reducing stress.
- More information about mindfulness can be found here: https://blackdoginstitute.org.au/docs/default-source/factsheets/mindfulnessineverdaylife.pdf
- And here: https://www.sane.org/information-stories/facts-and-guides/mindfulness

How To Use Destressify

- Download the **free version** of the app:
  from either the **Apple App Store** if you have an iPhone
  https://apps.apple.com/us/app/destressify-stress-relief/id751829934

  or **Google Play** if you have an Android phone
  https://play.google.com/store/apps/details?id=com.stressrefuge.destressify.free
- When you first open Destressify, there is the option of watching a 12-minute video about "what is stress?". It is not essential that you watch this, but feel free to do so if you wish. If you want to skip this video, tap on the "*Get Started*" button. You will need to create an account by providing an e-mail address, password, and other information. Follow the instructions to do this. If you're concerned about your data, read the Destressify privacy policy at https://www.destressify.com/privacy/
- When you have finished creating your profile, you are given the option to either "*View My Plan*" or "*View Practices*". The *View My Plan* option opens the core plan of mindfulness exercises that have already been selected for you. The *View Practices* option allows you to create your own plan. We recommend choosing the core plan and following their suggestions.

Figure 4b. Participant app information sheet for *Destressify*, page 2.

- After tapping on *View My Plan*, you can immediately begin your first mindfulness activity. You can also tap on "*View/Edit Schedule*" to see the names of future activities.

- In the top right corner of screen is the Menu folder. Tap on this to access the features of the app. Not all the features are available in this free version. If you want to access all the features, you need to download Destressify Pro at a cost, but this is not required for our research – just the free version of the app is needed.

- In the Menu, tap on the button "*Learn*" to read information on mindfulness and stress.

- In the Menu, tap on the button "*Practices*" to read information on the different types of activities available in the app.

- We would like you to use this app for at least 10 minutes per day, 5 days per week for at least 10 weeks. (We will tell you when the "official" use period has finished, but you will be able to keep using the app after this time if you want to.) If you want to use it for longer than 10 minutes per day and more than 5 days per week, this is OK – it's up to you. As long as you're using it for ***at least*** 10 minutes per day, 5 days per week.

- We will be asking you during the 10-week period how long you have approximately been using the app for.

- We will occasionally e-mail you links to some simple online questionnaires to fill out. These will mostly take less than 10 minutes. It would be appreciated if you could complete these as soon as possible after they arrive in your inbox.

- And don't forget to reply to our daily text messages. We will ask you how you're feeling that day, and you simply have to reply with a number from 0 to 10, with **0 indicating you are completely happy** and **10 indicating that you are in the worst mood possible**. At the end of the study, we will add up how many text message replies you have given us and we will give you $0.50 for each one we received.

If You Need Help

- In a mental health emergency, phone 000 or present at your local hospital emergency department.

- Lifeline: https://www.lifeline.org.au/ or **13 11 14**

- Beyond Blue: https://beyondblue.org.au/ or **1300 224 636**

- If you have any queries or concerns about the research project, please contact Jamie Marshall, Clinical Psychologist, by e-mail (jmarsh21@myune.edu.au) or phone [redacted]

Figure 5. Screenshots of the *Destressify* app that reveal the diversity in its content, including nutrition information, personalized plan, and the user's personal progress dashboard.

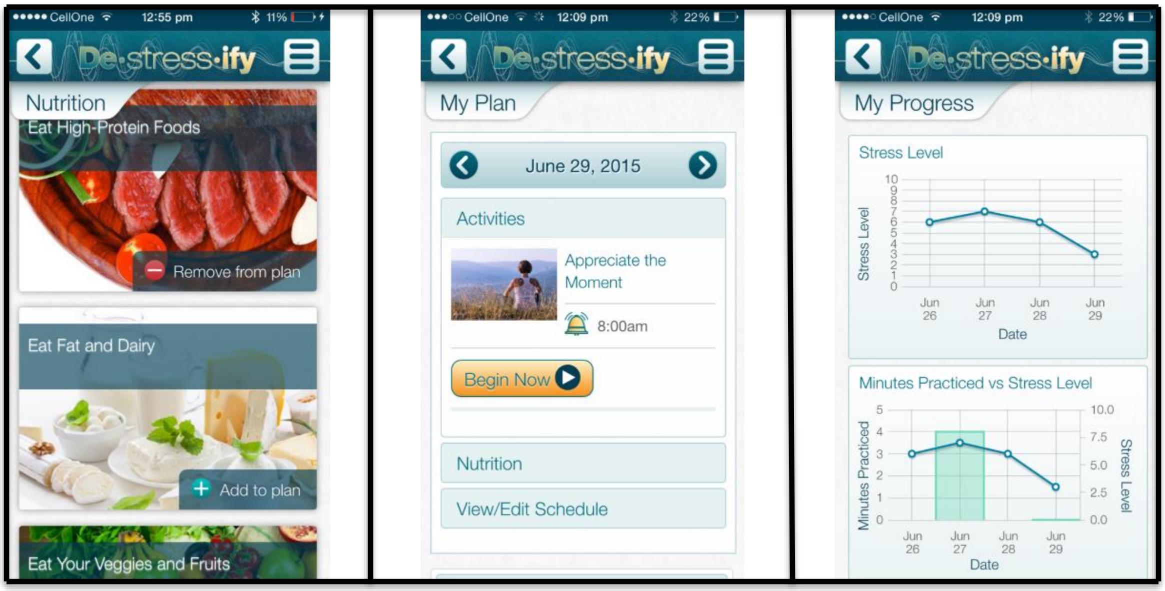

Figure 6a. Participant app information sheet for *MoodMission*, page 1.

APP INFORMATION

MoodMission

Background

- MoodMission was developed in Australia by a team of psychologists and mental health researchers at Monash University.
- More information about MoodMission can be found on their website: http://moodmission.com/.
- Research on this app has been published: Bakker, D., Kazantzis, N., Rickwood, D., & Rickard, N. (2018). A randomized controlled trial of three smartphone apps for enhancing public mental health. *Behavior Research and Therapy, 109*, 75-83.
- MoodMission uses an evidence-based technique called cognitive-behaviour therapy (CBT).
- More information about CBT can be found here: https://www.betterhealth.vic.gov.au/health/conditionsandtreatments/cognitive-behaviour-therapy.
- And here: https://au.reachout.com/articles/cognitive-behavioural-therapy.

How To Use MoodMission

- Download the app:
  from either the **Apple App Store** if you have an iPhone
  https://apps.apple.com/au/app/moodmission/id1140332763.

  or **Google Play** if you have an Android phone
  https://play.google.com/store/apps/details?id=com.moodmission.moodmissionapp.
- You will need to create an account by providing an e-mail address and password. Follow the instructions when you open the app for the first time after downloading it, and take the time to answer the questionnaire – it helps the app choose appropriate activities for you. If you're concerned about your data, read the MoodMission privacy policy at http://moodmission.com/privacy.
- MoodMission works by asking you questions about how you are feeling, and suggests all types of activities in response to how you answer the questions.
- MoodMission does not make you do just one thing, but always provides different options. If you do not like any of the listed options, you have the opportunity to ask MoodMission to suggest others.

Figure 6b. Participant app information sheet for *MoodMission*, page 2.

- If you are feeling acutely anxious or depressed, you can tap on "Other support options" at the bottom of the screen, and MoodMission provides emergency mental health support options you can immediately link in with.
- At the bottom left of screen is a "*Mission Log*" button. Tap on this to find a record of all your achievements, including the number of missions you have completed, and your rank. The more missions you complete, the higher your rank. You can also view other statistics here.
- At the bottom right of screen is the "*Expeditions*" button. Tap on this to find suggested programs for overcoming specific fears e.g. fear of public speaking, fear of flying etc.
- We would like you to use this app for at least 10 minutes per day, 5 days per week for at least 10 weeks. (We will tell you when the "official" use period has finished, but you will be able to keep using the app after this time if you want to.) If you want to use it for longer than 10 minutes per day and more than 5 days per week, this is OK – it's up to you. As long as you're using it for ***at least*** 10 minutes per day, 5 days per week.
- We will be asking you during the 10-week period how long you have approximately been using the app for.
- We will occasionally e-mail you links to some simple online questionnaires to fill out. These will mostly take less than 10 minutes. It would be appreciated if you could complete these as soon as possible after they arrive in your inbox.
- And don't forget to reply to our daily text messages. We will ask you how you're feeling that day, and you simply have to reply with a number from 0 to 10, with **0 indicating you are completely happy** and **10 indicating that you are in the worst mood possible**. At the end of the study, we will add up how many text message replies you have given us and we will give you $0.50 for each one we received.

If You Need Help

- In a mental health emergency, phone 000 or present at your local hospital emergency department.
- Lifeline: https://www.lifeline.org.au/ or **13 11 14**
- Beyond Blue: https://beyondblue.org.au/ or **1300 224 636**
- If you have any queries or concerns about the research project, please contact Jamie Marshall, Clinical Psychologist, by e-mail (jmarsh21@myune.edu.au) or phone [redacted]

Figure 7. Screenshots of the *MoodMission* app, highlighting its colorful interface, example of its emergency contact information page, example of a quick activity for the user to improve their mood, and an example of different activity options for the user.

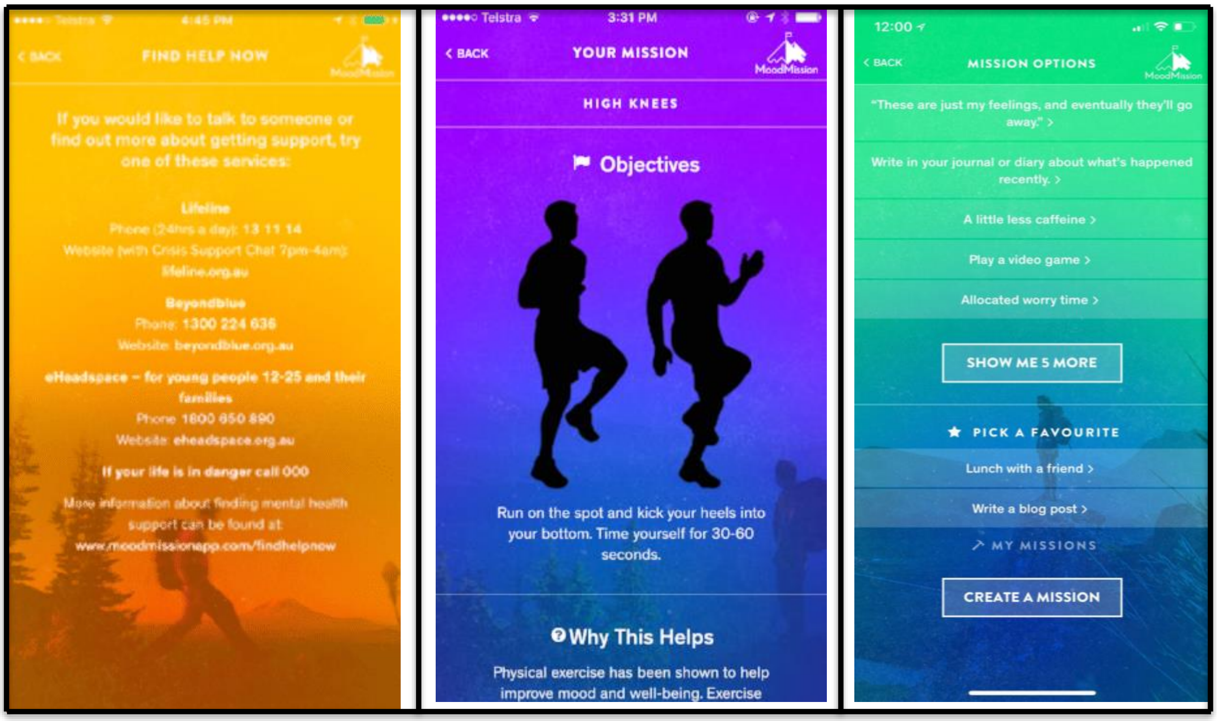

Figure 8a. Participant app information sheet for *Smiling Mind*, page 1.

APP INFORMATION

Smiling Mind

Background

- Smiling Mind was developed in Australia by mental health and meditation experts. It is a not-for-profit organisation.
- More information about Smiling Mind can be found on their website: https://www.smilingmind.com.au/
- Research on this app has been published: Flett, J. A. M., Hayne, H., Riordan, B. C., Thompson, L. M., & Conner, T. S. (2018). Mobile mindfulness meditation: A randomised controlled trial of the effect of two popular apps on mental health. *Mindfulness*, October.
- Smiling Mind is based on a technique called mindfulness, and has a meditation focus.
- More information about mindfulness can be found here: https://blackdoginstitute.org.au/docs/default-source/factsheets/mindfulnessineverdaylife.pdf
- And here: https://www.sane.org/information-stories/facts-and-guides/mindfulness

How To Use Smiling Mind

- Download the app:
  from either the **Apple App Store** if you have an iPhone
  https://itunes.apple.com/au/app/smiling-mind/id560442518?mt=8

  or **Google Play** if you have an Android phone
  https://play.google.com/store/apps/details?id=com.smilingmind.app&hl=en_AU
- You will need to create an account by providing an e-mail address and password. Follow the instructions when you open the app for the first time after downloading it. If you're concerned about your data, read the Smiling Mind privacy policy at https://www.smilingmind.com.au/privacy-policy
- After you have created an account, tap on the "*All Programs*" tab at the top right of screen. This will give you a list of all the programs available within the app. We recommend that you use the "*Adult Programs*" to start with because this consists of the greatest variety of activities.
- Once in "*Adult Programs*", feel free to scroll down the page and start using any of the programs by tapping on it. (Our recommendation is to start at the top first, and then work your way down through the programs.)

Figure 8b. Participant app information sheet for *Smiling Mind*, page 2.

- You can create a list of your favourite activities by adding them to your favourites folder. You simply tap on the activity, and when it opens, tap on the love heart in the top right hand corner. If you want to access something you have saved in favourites, look along the bottom of the screen and you should see the "*Favourites*" folder towards the right.

- There is a simple dashboard that allows you to see how many sessions you have completed and how much time you have spent meditating. Just tap on the "*Dashboard*" tab at the bottom of screen.

- To select a new program to do, first tap on "*My Programs*" at the bottom left of screen, and then tap on any of the three tabs at the top of the screen that say: "*My Programs*", "*My Sessions*", or "*All Programs*".

- We would like you to use this app for at least 10 minutes per day, 5 days per week for at least 10 weeks. (We will tell you when the "official" use period has finished, but you will be able to keep using the app after this time if you want to.) If you want to use it for longer than 10 minutes per day and more than 5 days per week, this is OK – it's up to you. As long as you're using it for ***at least*** 10 minutes per day, 5 days per week.

- We will be asking you during the 10-week period how long you have approximately been using the app for.

- We will occasionally e-mail you links to some simple online questionnaires to fill out. These will mostly take less than 10 minutes. It would be appreciated if you could complete these as soon as possible after they arrive in your inbox.

- And don't forget to reply to our daily text messages. We will ask you how you're feeling that day, and you simply have to reply with a number from 0 to 10, with **0 indicating you are completely happy** and **10 indicating that you are in the worst mood possible**. At the end of the study, we will add up how many text message replies you have given us and we will give you $0.50 for each one we received.

If You Need Help

- In a mental health emergency, phone 000 or present at your local hospital emergency department.

- Lifeline: https://www.lifeline.org.au/ or **13 11 14**

- Beyond Blue: https://beyondblue.org.au/ or **1300 224 636**

- If you have any queries or concerns about the research project, please contact Jamie Marshall, Clinical Psychologist, by e-mail (jmarsh21@myune.edu.au) or phone [redacted]

Figure 9. Screenshots of the *Smiling Mind* app, with examples of its different mindfulness activities, its brief survey before and after meditation activities (which it uses to track progress over time), and the screen that appears when listening to one of its activities.

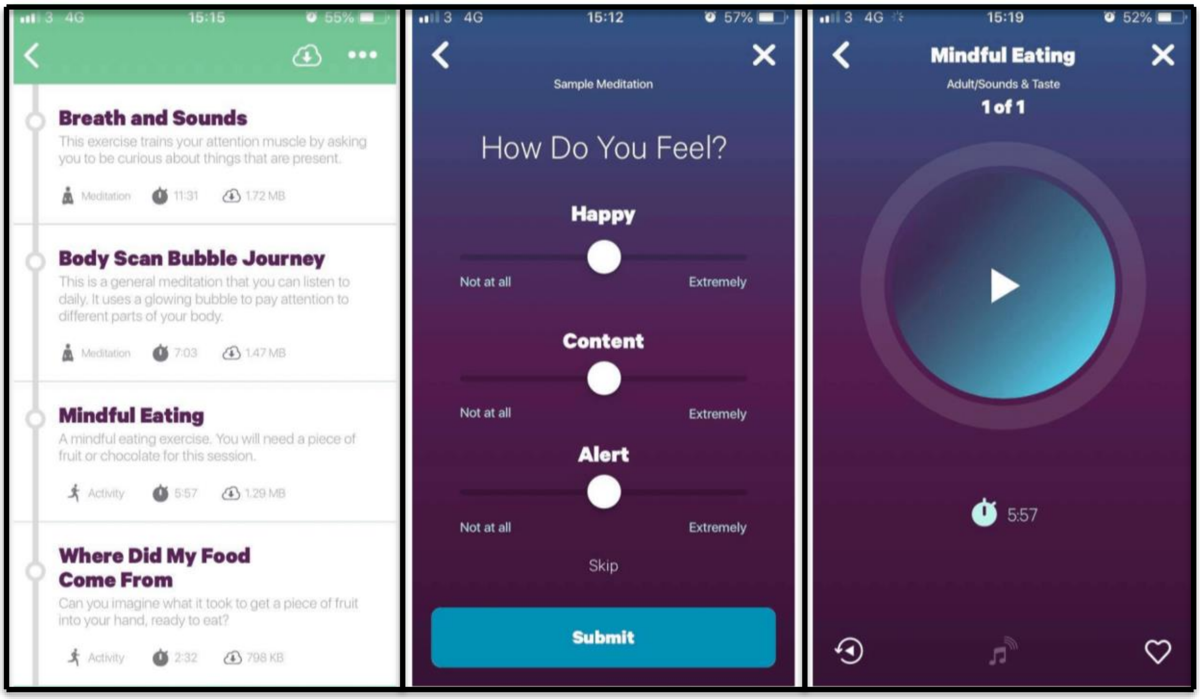

Figure 10a. Participant app information sheet for *MindShift*, page 1.

APP INFORMATION

MindShift CBT

Background

- MindShift was developed in Canada by a not-for-profit mental health support organisation.
- More information about MindShift can be found on their website: https://anxietycanada.com/resources/mindshift-cbt/
- Research on this app has been published: Paul, A. M., & Fleming, C. J. E. (2019). Anxiety management on campus: An evaluation of a mobile health intervention. *Journal of Technology in Behavioral Science*, 4(1), 58-61.
- MindShift uses an evidence-based technique called cognitive-behaviour therapy (CBT). Although its focus is anxiety, the techniques can be applied to depression as well.
- More information about CBT can be found here: https://www.betterhealth.vic.gov.au/health/conditionsandtreatments/cognitive-behaviour-therapy.
- And here: https://au.reachout.com/articles/cognitive-behavioural-therapy.

How To Use MindShift

- Download the app:
  from either the **Apple App Store** if you have an iPhone
  https://apps.apple.com/au/app/mindshift-cbt-anxiety-canada/id634684825

  or **Google Play** if you have an Android phone
  https://play.google.com/store/apps/details?id=com.bstro.MindShift&hl=en
- You will need to create an account by providing an e-mail address, password, and other information. If you're concerned about your data, read the MindShift privacy policy at: https://anxietycanada.com/mindshift-cbt-privacy-policy/
- After you have created an account, the app opens to its home screen (which you can access at any time by tapping on the "*Home*" button at the bottom left of screen). The Home screen allows you to choose how you are feeling today, and what type of anxiety you suffer from.
- Tapping on a type of anxiety takes you to information about that type of worry. When you scroll down the anxiety screen, the app allows you to select categories within that type of anxiety which lists some of the more common unhelpful thoughts associated with it.

Figure 10b. Participant app information sheet for *MindShift*, page 2.

- We recommend that you start by scrolling down the home page until you come to "*Tools*". Tap on the button, "*Healthy Thinking*", then tap on "*Thought Journal*", and then tap on "*Add New +*". This will get you started using one of the main functions of the app: coming up with more helpful thoughts when you are feeling anxious and/or depressed due to thinking negatively.

- You can tap on "*Learn*" at the bottom of screen any time to read more information about anxiety and CBT. You can tap on "*Goals*" at the bottom of screen any time to set goals you would like to achieve.

- If you would like some suggestions from the app about things you can do quickly to help ease acute anxiety or depression symptoms, tap on the blue smiley face at the bottom centre of screen.

- We would like you to use this app for at least 10 minutes per day, 5 days per week for at least 10 weeks. (We will tell you when the "official" use period has finished, but you will be able to keep using the app after this time if you want to.) If you want to use it for longer than 10 minutes per day and more than 5 days per week, this is OK – it's up to you. As long as you're using it for ***at least*** 10 minutes per day, 5 days per week.

- We will be asking you during the 10-week period how long you have approximately been using the app for.

- We will occasionally e-mail you links to some simple online questionnaires to fill out. These will mostly take less than 10 minutes. It would be appreciated if you could complete these as soon as possible after they arrive in your inbox.

- And don't forget to reply to our daily text messages. We will ask you how you're feeling that day, and you simply have to reply with a number from 0 to 10, with **0 indicating you are completely happy** and **10 indicating that you are in the worst mood possible**. At the end of the study, we will add up how many text message replies you have given us and we will give you $0.50 for each one we received.

If You Need Help

- In a mental health emergency, phone 000 or present at your local hospital emergency department.

- Lifeline: https://www.lifeline.org.au/ or **13 11 14**

- Beyond Blue: https://beyondblue.org.au/ or **1300 224 636**

- If you have any queries or concerns about the research project, please contact Jamie Marshall, Clinical Psychologist, by e-mail (jmarsh21@myune.edu.au) or phone [redacted]

Figure 11. Screenshots of the *MindShift* app, highlighting its bright interface, home screen, example of psychoeducation regarding helpful and unhelpful thinking, and symptom monitoring tools.

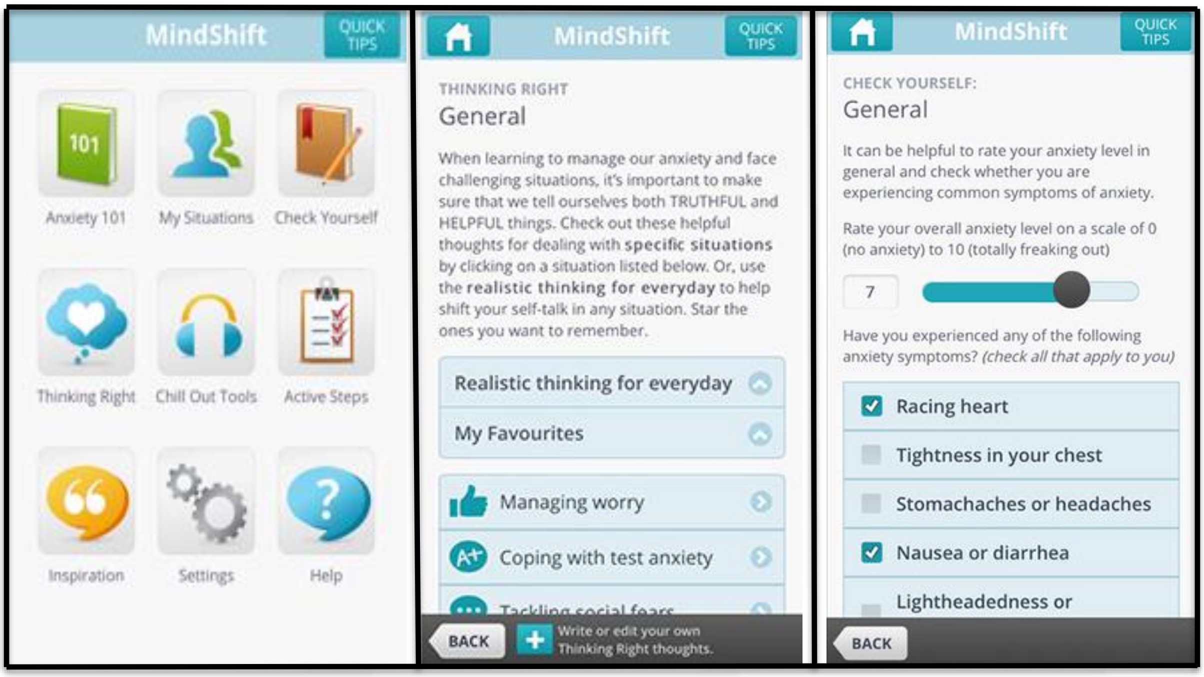

Figure 12a. Participant app information sheet for *SuperBetter*, page 1.

APP INFORMATION

SuperBetter

Background

- SuperBetter was developed in the United States by game designer Jane McGonigal, with assistance from researchers at Stanford University and the University of Pennsylvania. She gives a talk that provides some background on her approach at: https://www.youtube.com/watch?v=irsTFdCtcuQ.
- More information about SuperBetter can be found on their website: https://www.superbetter.com/.
- Research on this app has been published: Roepke, A. M., Jaffee, S. R., Riffle, O. M., McGonigal, J., Broome, R., & Maxwell, B. (2015). Randomized controlled trial of SuperBetter, a smartphone-based/Internet-based self-help tool to reduce depressive symptoms. *Games for Health Journal, 4*(3), 235-246.
- SuperBetter uses evidence-based techniques from positive psychology and neuroscience (neuroplasticity).
- More information about positive psychology can be found here: https://www.blackdoginstitute.org.au/docs/default-source/factsheets/positivepsychology.pdf.
- More information about neuroscience, neuroplasticity, and mental health can be found here: https://positivepsychology.com/neuroplasticity/.

How To Use SuperBetter

- Download the app:
  from either the **Apple App Store** if you have an iPhone
  https://apps.apple.com/au/app/superbetter/id536634968.

  or **Google Play** if you have an Android phone
  https://play.google.com/store/apps/details?id=com.superbetter.paid&hl=en.
- You will need to create an account by providing an e-mail address and password. Follow the instructions when you open the app for the first time after downloading it, and take the time to answer the questions – it helps the app choose appropriate activities for you. If you're concerned about your data, read the SuperBetter privacy policy at https://www.superbetter.com/terms (note that you have to scroll down about halfway to get to the privacy section).
- A good place to start with SuperBetter is to tap on the "More" button at the bottom right of screen, then tap on "FAQ", and read the entire FAQ section. It provides an excellent background to how SuperBetter works and will get you ready to start "playing" immediately.

Figure 12b. Participant app information sheet for *SuperBetter*, page 2.

- SuperBetter uses the idea of playing a video game and works by getting you to complete "quests". Quests are activities designed to get you closer to achieving your specific goals.
- "Power-ups" are smaller activities designed to make you feel better and improve your mood quickly. Part of the game of SuperBetter is to complete as many power-ups and quests as you can.
- SuperBetter also has "bad guys" and "allies". Bad guys are things that get in the way of achieving your goals, such as anxiety or depression. Allies are people who support you on your journey, such as a therapist, family member, or friend. You can send details about "epic wins" and other achievements to your allies, and they can also send you messages of support.
- After you have read the FAQ section, we recommend that you tap on the "*SB To do*" button at the bottom centre of screen, then tap the "*Do 3 Quests*" banner, then tap the "*Hero, Start Here!*" button. Follow the instructions about setting up your first quest.
- We would like you to use this app for at least 10 minutes per day, 5 days per week for at least 10 weeks. (We will tell you when the "official" use period has finished, but you will be able to keep using the app after this time if you want to.) If you want to use it for longer than 10 minutes per day and more than 5 days per week, this is OK – it's up to you. As long as you're using it for ***at least*** 10 minutes per day, 5 days per week.
- We will be asking you during the 10-week period how long you have approximately been using the app for.
- We will occasionally e-mail you links to some simple online questionnaires to fill out. These will mostly take less than 10 minutes. It would be appreciated if you could complete these as soon as possible after they arrive in your inbox.
- And don't forget to reply to our daily text messages. We will ask you how you're feeling that day, and you simply have to reply with a number from 0 to 10, with **0 indicating you are completely happy** and **10 indicating that you are in the worst mood possible**. At the end of the study, we will add up how many text message replies you have given us and we will give you $0.50 for each one we received.

If You Need Help

- In a mental health emergency, phone 000 or present at your local hospital emergency department.
- Lifeline: https://www.lifeline.org.au/ or **13 11 14**
- Beyond Blue: https://beyondblue.org.au/ or **1300 224 636**
- If you have any queries or concerns about the research project, please contact Jamie Marshall, Clinical Psychologist, by e-mail (jmarsh21@myune.edu.au) or phone [redacted]

Figure 13. Screenshots of the *SuperBetter* app, highlighting its colorful interface, example to-do list, example power-ups screen, and example reward notification.

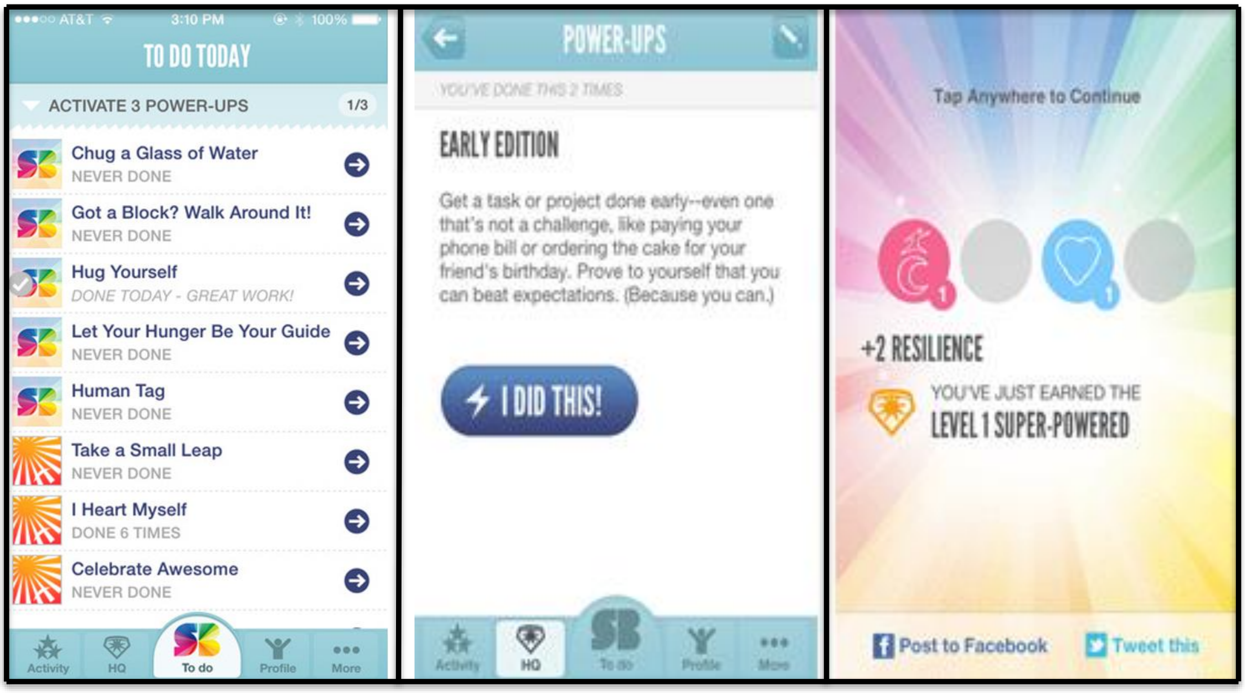

Table 1. Characteristics summary of each mental health app in the present study.

App name	Theoretical framework(s)	Corporate governance	Previous research	Country of origin
Destressify	Mindfulness	Private corporation	Lee, Jung [1]	United States of America
MoodMission	CBT	Private corporation	Bakker, Rickard [2]	Australia
Smiling Mind	Mindfulness	Not-for-profit	Flett, Hayne, Riordan, Thompson, Conner [3]	Australia
MindShift	CBT	Not-for-profit	Paul, Fleming [4]	Canada
SuperBetter	CBT / Positive Psychology / Neuroplasticity	Private corporation	Roepke, Jaffee, Riffle, McGonigal, Broome, Maxwell [5]	United States of America

Table 2. Links to professional independent reviews for mental health apps in the present study.

App name	Link to PsyberGuide review	Link to other independent review
Destressify	None found	None found
MoodMission	https://psyberguide.org/apps/moodmission/	https://au.reachout.com/tools-and-apps/moodmission
Smiling Mind	https://psyberguide.org/apps/smiling-mind/	https://au.reachout.com/tools-and-apps/smiling-mind
MindShift	https://psyberguide.org/apps/mindshift/	https://www.healthnavigator.org.nz/apps/m/mindshift-app/
SuperBetter	https://psyberguide.org/apps/superbetter/	https://au.reachout.com/tools-and-apps/superbetter

Table 3. Summary details of previous research on the apps used in the present study.

Article authors (*app name*)	Design / methodology	Sample type	Study focus	Outcome measures used
Lee, Jung [1] (*Destressify*)	4 weeks app use – 5 days per week. Participants told to use the “Core Plan” on the app and told not to engage with other features of the app. Intervention app is based on mindfulness. Control group was waitlisted	American undergraduate students	Anxiety, depression, stress	State-Trait Anxiety Inventory; Quick Inventory of Depressive Symptomatology Self-Report; Perceived Stress Scale (PSS)
Bakker, Rickard [2] (*MoodMission*)	30 days total app use with four push notifications sent to participants during this period reminding them to engage with the app. Intervention app is based on CBT	Australian community sample of individuals who first downloaded the app and then agreed to be in the research	Anxiety, depression	Generalized Anxiety Disorder Scale 7-Item; Patient Health Questionnaire 9-Item; Warwick Edinburgh Mental Well-Being Scale
Flett, Hayne, Riordan, Thompson, Conner [3] (*Smiling Mind*)	40 days total app use – 10 mins per day for the first 10 days, then use the app at participant discretion for remaining 30 days. The app intervention is based on mindfulness. RCT design with control group using an app named Evernote to test for the digital placebo effect	New Zealand undergraduate students	Anxiety, depression, stress, flourishing	Hospital Anxiety and Depression Scale-Anxiety Subscale; Center for Epidemiological Studies Depression Scale (CES-D); PSS; Flourishing Scale
Paul, Fleming [4] (*MindShift*)	3 weeks app use – minimum of 15 mins per day, 5 days per week. The app has an anxiety focus and is underpinned by CBT. Participants were only included if they were not receiving any other treatments for anxiety. Participants were given a tutorial on using the app	Canadian undergraduate students	Focus was on anxiety, but also examined depression	Patient Health Questionnaire (PHQ)-15; PHQ-Panic; Generalized Anxiety Disorder Scale-7 (GAD-7); PHQ-9
Roepke, Jaffee, Riffle, McGonigal, Broome, Maxwell [5] (*SuperBetter*)	App use for 10 mins per day for 1 month using an RCT design with a waitlist control group. Sample was described as “motivated” individuals who already had an interest in managing their mental health with an app because they had first downloaded the app before being recruited to the trial. App is based on CBT / positive psychology / neuroplasticity	Americans who downloaded the app and self-report measure indicated significant depression symptoms	Anxiety, depression, life satisfaction	GAD-7; CES-D; Satisfaction With Life Scale

Table 4. Summary of statistics of previous research on the apps used in the present study.

Article authors (*app name*)	Intervention group N	Control group N	Mean age (years)	*P*-value	Effect size
Lee, Jung [1] (*Destressify*)	77	86	20.6	Anxiety = .02*<br>Depression = .09<br>Stress = .06	η^2_p = .05 (anxiety)<br>η^2_p = .02 (depression)<br>η^2_p = .02 (stress)
Bakker, Rickard [2] (*MoodMission*)	617	None	26.9	Anxiety (severe clinical) = .05*<br>Depression (severe clinical) = .01*	β (Anxiety – severe clinical – coping self-efficacy mediator) = - .378<br>β (Depression – severe clinical – coping self-efficacy mediator) = - .386
Flett, Hayne, Riordan, Thompson, Conner [3] (*Smiling Mind*)	58	67	20.1	Anxiety = < .05*<br>Depression = > .05<br>Stress = > .05<br>Flourishing = > .05	Cohen's d = .29 (anxiety),<br>Cohen's d = .13 (depression),<br>Cohen's d = .18 (stress),<br>Cohen's d = .01 (flourishing)
Paul, Fleming [4] (*MindShift*)	16	None	19.8	Somatic anxiety = < .01*<br>General anxiety = < .01*<br>Panic = .99<br>Depression = < .01*	η = .34 (somatic anxiety)<br>η = .51 (general anxiety)<br>η = .00 (panic)<br>η = .30 (depression)
Roepke, Jaffee, Riffle, McGonigal, Broome, Maxwell [5] (*SuperBetter*)	190 (97 in regular app group, and 93 in "enhanced" app group)	93	Not given	Regular app anxiety = < .001*<br>Enhanced app anxiety = .01*<br>Regular app depression = < .001*<br>Enhanced app depression = .01*<br>Regular app life satisfaction = .007*<br>Enhanced app life satisfaction = < .001*	Cohen's d regular app = 0.92 (depression)<br>Cohen's d enhanced app = 0.43 (depression)<br>(Note: Individual effect sizes for other outcomes not given in article, but it was stated that they were between 0.43 and 1.36.)

* Indicated as statistically significant in the article.

Research Summary

Lee, Jung [1] found statistically significant reductions in anxiety from pre- to post-treatment, but found no significant change in depression or stress; Bakker, Rickard [2] recorded statistically significant improvements in anxiety and depression in “nonclinical” and “severe” participants (as determined by their self-report measures at study commencement), with no such improvements in “moderate” participants; Flett, Hayne, Riordan, Thompson, Conner [3] found statistically significant reductions in anxiety symptoms, but not depression; Paul, Fleming [4] found statistically significant reductions in somatic and generalized anxiety, and depression, but not for panic; and Roepke, Jaffee, Riffle, McGonigal, Broome, Maxwell [5] obtained statistically significant reductions in anxiety and depression compared to waitlist.

References

1. Lee RA, Jung ME. Evaluation of an mHealth app (Destressify) on university students' mental health: Pilot trial. *JMIR Mental Health.* 2018;5(1):e2; doi: 10.2196/mental.8324.
2. Bakker D, Rickard N. Engagement with a cognitive behavioural therapy mobile phone app predicts changes in mental health and wellbeing: MoodMission. *Aust Psychol.* 2019;54(4):245-260; doi: 10.1111/ap.12383.
3. Flett JAM, Hayne H, Riordan BC, Thompson LM, Conner TS. Mobile mindfulness meditation: A randomised controlled trial of the effect of two popular apps on mental health. *Mindfulness.* 2019;10(5):863-876; doi: 10.1007/s12671-018-1050-9.
4. Paul AM, Fleming CJE. Anxiety management on campus: An evaluation of a mobile health intervention. *Journal of Technology in Behavioral Science.* 2019;4(1):58-61; doi: 10.1007/s41347-018-0074-2.
5. Roepke AM, Jaffee SR, Riffle OM, McGonigal J, Broome R, Maxwell B. Randomized controlled trial of SuperBetter, a smartphone-based/Internet-based self-help tool to reduce depressive symptoms. *Games For Health Journal.* 2015;4(3):235-246; doi: 10.1089/g4h.2014.0046.
