# Supplementary material for: Effectiveness of Using Mental Health Mobile Apps as Digital Antidepressants for Reducing Anxiety and Depression: Protocol for a Multiple Baseline Across-Individuals Design
Source: JMIR Res Protoc. 2020 Jul 5;9(7):e17159. doi: 10.2196/17159 (PMC7381081; doi:10.2196/17159)
Supplement: Multimedia Appendix 3 [file resprot_v9i7e17159_app3.pdf]

Multimedia Appendix 3

Forms and Measures Used in the Present Study

In a mental health emergency,
phone Lifeline on 13 11 14 or the Suicide Callback Service on 1300 659 467,
or present at the emergency department of your nearest hospital

Information for Participants

INFORMATION FOR PARTICIPANTS

I wish to invite you to participate in my research project, described below.

My name is Jamie Marshall and I am conducting this research as part of my PhD in the School of Psychology at the University of New England. My supervisors are Prof Debra Dunstan and Dr Warren Bartik.

**Research Project Title: I bet there's an app for that: Using mobile mental health apps for reducing anxiety and depression**
**Aim of the Research**: The research aims to explore the effectiveness of certain mobile mental health apps in reducing symptoms of anxiety and depression.
**Survey**: Following this information sheet, you will be asked to take a survey that will take less than 30 minutes to complete. Some time after you have completed and submitted the survey, you will be sent an e-mail or text message with further information about the study.
**Confidentiality**: Any personal details gathered in the course of the study will remain

confidential. No individual will be identified by name in any publication of the results. All names will be replaced by pseudonyms or numbers; this will ensure your anonymity. If you agree, I would like to quote some of your responses. This will also be done in a way to ensure that you are not identified.

Participation is Voluntary: Please understand that your involvement in this study is voluntary and I respect your right to stop participating in the study at any time without consequence and without needing to provide an explanation. If you choose to remain in the study for the duration of the data gathering phase, this will likely take approximately 18 weeks. During this time, you will be asked to use a mobile app as instructed. Other than completing some questionnaires at four different time points, you will also be asked to provide a daily reply text message with a number out of 10 indicating your mood for that day. For each daily text message you send in, you will receive a payment of $0.50 cents, payable at the conclusion of the study. We would also like to contact you six months after your involvement has finished with another questionnaire.

Questions: Questions contained in the surveys will concern mental health, specifically anxiety and depression, and your use of mobile apps to reduce symptoms of these issues.

Use of Information: I will use information from the surveys as part of my doctoral thesis, which I expect to complete in December 2020. Information from the surveys may also be used in academic journal articles and conference presentations before and after this date. At all times, I will safeguard your identity by presenting the information in a way that will not allow you to be identified.

Upsetting Issues: If this research raises any personal or upsetting issues, please contact Lifeline on 13 11 14, or your local Community Health Centre.

Storage of Information: All hardcopy notes or information generated by this study will be scanned, uploaded, and saved with all other electronic data and will be kept on Cloud.UNE, UNE's centrally managed cloud server accessible only by the research team. It

will also be kept on a password protected computer in the same location that only the research team will have access to.

Disposal of Information: All the data collected in this research will be kept indefinitely on UNE's centrally managed research data storage facility, Cloud.UNE. All information stored in local computer hard drives will be deleted after the conclusion of the research.

Requirements: To participate in this research, you are required to have access to a smartphone and/or tablet device, be 18 years of age or older, and have previously been diagnosed with an anxiety and/or depression condition by a qualified health professional. If your mental health condition and/or history includes psychotic episodes, this research project is not suitable for you.

Approval: This project has been approved by the Human Research Ethics Committee of the University of New England (Approval No. HE19-186, Valid to 01/11/2020).

Contact Details for Researchers:

Feel free to contact me with any questions about this research by email at **jmarsh21@myune.edu.au** or by phone during business hours on [redacted]

You may also contact my supervisors.

My Principal supervisor's name is Prof Debra Dunstan and she can be contacted by email at [redacted] or by phone on [redacted].

My Co-supervisor's name is Dr Warren Bartik and he can be contacted by email at [redacted] or by phone on [redacted].

Complaints: Should you have any complaints concerning the manner in which this research is conducted, please contact:

Mrs Jo-Ann Sozou

Research Ethics Officer

Research Services

University of New England, Armidale, NSW 2351

Tel: (02) 6773 3449 Email: humanethics@une.edu.au

Thank you for considering this request and I look forward to further contact with you.

Regards,

JAMIE MARSHALL

Clinical Psychologist & PhD Candidate

M.Psych. (clinical), B.A. (Hons.) (Psych.), Grad. Dip. Soc. Sci., MAPS FCCLP

AHPRA Registration No. PSY0001685145

Participant Consent

CONSENT:

I have read the Information For Participants;

I agree to participate in this activity, knowing that I may withdraw at any time;

I agree that research data gathered for this study may be quoted and published without identifying me; and

I am 18 years of age or older.

- Yes, I consent to participate in this research project.
- No, I do not wish to participate in this research project.

Personal Details / Demographic Information 1

We would like to ask some personal questions that may improve our ability to understand the findings from this research. Remember, your name will never be matched to this information.

What is your age (in years only)?

What is your gender?

- Male
- Female
- Other

Where do you live?

- In a major city
- In a large regional city/town
- In a rural / remote area

Please indicate the highest level of education you have completed:

- Primary (Year 6 or below)
- Secondary (Years 7 - 10)
- Secondary (Years 11 or 12)
- University degree
- Other tertiary (e.g. TAFE)

Personal Details / Demographic Information 2

Continued ...

Your occupation (if not being paid to work, please write "not in paid work"):

If in paid employment, how many hours do you work a week?

- ○ Less than 30 hours
- ○ 30 hours or more
- ○ Not in paid work

Approximately how much money do you earn a week after tax? (Including any government payments you receive)

- ○ Less than $300
- ○ $301 - $600
- ○ $601 - $1000
- ○ More than $1000
- ○ Prefer not to say

Mental Health Information

We would now like to ask some questions related to your health, and specifically your mental health. These questions are very important to this research and we ask that you answer as honestly as you can even though much of this information is sensitive. Again, remember that your name will not be matched to any of this information.

In the past, you have previously been diagnosed with (select more than one if applicable):

- ☐ Depression
- ☐ Anxiety (including panic attacks, posttraumatic stress disorder, obsessive-compulsive disorder, social anxiety, and phobias) - Please state what type of anxiety you suffer from.
- ☐ Other (please type in any other mental health condition you have been diagnosed with)

Approximately how long have you had anxiety and/or depression?

- ◯ Less than a year
- ◯ 1-5 years
- ◯ 6-10 years
- ◯ 11 years or longer

Are you currently seeing a psychiatrist, psychologist, counsellor, or other mental health professional for counselling related to your anxiety and/or depression?

- ☐ Psychiatrist
- ☐ Psychologist
- ☐ Counsellor
- ☐ Other mental health professional
- ☐ Not currently receiving counselling

Are you currently on any type of medication for your anxiety and/or depression?

- ◯ Yes
- ◯ No

If yes, what type of medication is it? (If you know the name of your medication but you are not sure which category it belongs to, write the name of it next to "Other")

- ☐ Don't know
- ☐ I've never been on medication for my anxiety and/or depression
- ☐ Antidepressant
- ☐ Benzodiazepine / tranquiliser

- ☐ Antipsychotic
- ☐ Mood stabiliser
- ☐ Other (if known, please type in the name of the medication if it does not fit into any of the above categories)

What self-help strategies do you currently practice to help manage your symptoms of anxiety and/or depression? (Select all that apply)

- ☐ Meditation / mindfulness
- ☐ Physical exercise
- ☐ Dietary considerations
- ☐ Abstain from drinking alcohol
- ☐ Increase my social contact and activities with other people
- ☐ Engage in a hobby
- ☐ Other (please tell us)

General Health & Other Information

Continued ...

Do you currently suffer from any other chronic medical condition (e.g. asthma, diabetes etc.)?

- ○ No
- ○ Yes (please tell us what other medical conditions you have)

On average, how many hours of sleep would you get each night?

- ○ Less than 4 hours
- ○ 4-6 hours
- ○ 7-8 hours

- ○ More than 8 hours

Overall, how would you rate your physical health?

- ○ Excellent
- ○ Good
- ○ Average
- ○ Poor
- ○ Terrible

In your opinion, which of the following can cause anxiety and/or depression? (You can select more than one)

- ☐ Don't know
- ☐ Genetics (family history)
- ☐ Weak character
- ☐ Abnormal brain chemistry (chemicals in the brain not working properly)
- ☐ The weather (people in cold, dark places get more depressed than people in warmer, bright places)
- ☐ Major catastrophic event (e.g. death of a loved one, natural disaster, being the victim of a crime etc.)
- ☐ Other (please type in anything else that may cause anxiety and/or depression) __________

How would you rate the following treatment options for anxiety and/or depression?

	Helpful	Harmful	Don't Know
GP / Family Doctor	○	○	○
Pharmacist / Chemist	○	○	○
Counsellor or Social Worker	○	○	○

	Helpful	Harmful	Don't Know
Telephone counselling service (such as Lifeline)	○	○	○
Webchat / website counselling service	○	○	○
Psychiatrist	○	○	○
Psychologist	○	○	○
Close family and friends	○	○	○
Naturopath / herbalist	○	○	○
The clergy, a minister, or a priest	○	○	○

Please respond to the following statements by checking the appropriate response:

	Strongly disagree	Somewhat disagree	Neither agree nor disagree	Somewh agree
My psychological health is likely to improve as a result of participating in this research	○	○	○	○
I am ready to work on changing my psychological health for the better	○	○	○	○
I am motivated to do what the mobile app suggests is good for my psychological health	○	○	○	○

What brand of smartphone do you use?

- ○ Apple iPhone
- ○ Samsung
- ○ Google Pixel
- ○ Oppo
- ○ Huawei

- Nokia
- LG
- Other (please tell us what type of phone you have if it's not in the above list)

How would you rate your abilities to use the functions of your smartphone?

Terrible	Poor	Average	Good	Excellent
○	○	○	○	○

How would you rate your abilities to engage with and use other types of technology generally?

Terrible	Poor	Average	Good	Excellent
○	○	○	○	○

Have you previously downloaded a mobile app to help your anxiety and/or depression?

- No
- Yes (please tell us the name of any apps that you remember downloading)

If you have previously downloaded an app to help your anxiety and/or depression, was it helpful?

- No
- Yes
- I've never downloaded an app to help my anxiety and/or depression

Technology has the potential to help people manage their anxiety and/or depression.

Strongly disagree	Somewhat disagree	Neither agree nor disagree	Somewhat agree	Strongly agree
○	○	○	○	○

Powered by Qualtrics

SUDS

HOW DO I FEEL TODAY?

- ○ **0** ..... No distress
- ○ **1**
- ○ **2** ..... Minimal distress
- ○ **3**
- ○ **4**
- ○ **5** ..... Moderate distress
- ○ **6**
- ○ **7**
- ○ **8** ..... Very distressed
- ○ **9**
- ○ **10** ..... Worst distress

Powered by Qualtrics

DASS21

Name: Date:

Please read each statement and circle a number 0, 1, 2 or 3 which indicates how much the statement applied to you **over the past week**. There are no right or wrong answers. Do not spend too much time on any statement.

The rating scale is as follows:

0 Did not apply to me at all
1 Applied to me to some degree, or some of the time
2 Applied to me to a considerable degree or a good part of time
3 Applied to me very much or most of the time

1 (s)	I found it hard to wind down	0	1	2	3
2 (a)	I was aware of dryness of my mouth	0	1	2	3
3 (d)	I couldn't seem to experience any positive feeling at all	0	1	2	3
4 (a)	I experienced breathing difficulty (e.g. excessively rapid breathing, breathlessness in the absence of physical exertion)	0	1	2	3
5 (d)	I found it difficult to work up the initiative to do things	0	1	2	3
6 (s)	I tended to over-react to situations	0	1	2	3
7 (a)	I experienced trembling (e.g. in the hands)	0	1	2	3
8 (s)	I felt that I was using a lot of nervous energy	0	1	2	3
9 (a)	I was worried about situations in which I might panic and make a fool of myself	0	1	2	3
10 (d)	I felt that I had nothing to look forward to	0	1	2	3
11 (s)	I found myself getting agitated	0	1	2	3
12 (s)	I found it difficult to relax	0	1	2	3
13 (d)	I felt down-hearted and blue	0	1	2	3
14 (s)	I was intolerant of anything that kept me from getting on with what I was doing	0	1	2	3
15 (a)	I felt I was close to panic	0	1	2	3
16 (d)	I was unable to become enthusiastic about anything	0	1	2	3
17 (d)	I felt I wasn't worth much as a person	0	1	2	3
18 (s)	I felt that I was rather touchy	0	1	2	3
19 (a)	I was aware of the action of my heart in the absence of physical exertion (e.g. sense of heart rate increase, heart missing a beat)	0	1	2	3
20 (a)	I felt scared without any good reason	0	1	2	3
21 (d)	I felt that life was meaningless	0	1	2	3

DASS-21 Scoring Instructions

The DASS-21 should not be used to replace a face to face clinical interview. If you are experiencing significant emotional difficulties you should contact your GP for a referral to a qualified professional.

Depression, Anxiety and Stress Scale - 21 Items (DASS-21)

The Depression, Anxiety and Stress Scale - 21 Items (DASS-21) is a set of three self-report scales designed to measure the emotional states of depression, anxiety and stress.

Each of the three DASS-21 scales contains 7 items, divided into subscales with similar content. The depression scale assesses dysphoria, hopelessness, devaluation of life, self-deprecation, lack of interest / involvement, anhedonia and inertia. The anxiety scale assesses autonomic arousal, skeletal muscle effects, situational anxiety, and subjective experience of anxious affect. The stress scale is sensitive to levels of chronic non-specific arousal. It assesses difficulty relaxing, nervous arousal, and being easily upset / agitated, irritable / over-reactive and impatient. Scores for depression, anxiety and stress are calculated by summing the scores for the relevant items.

The DASS-21 is based on a dimensional rather than a categorical conception of psychological disorder. The assumption on which the DASS-21 development was based (and which was confirmed by the research data) is that the differences between the depression, anxiety and the stress experienced by normal subjects and clinical populations are essentially differences of degree. The DASS-21 therefore has no direct implications for the allocation of patients to discrete diagnostic categories postulated in classificatory systems such as the DSM and ICD.

Recommended cut-off scores for conventional severity labels (normal, moderate, severe) are as follows:

NB Scores on the DASS-21 will need to be multiplied by 2 to calculate the final score.

	**Depression**	**Anxiety**	**Stress**
Normal	0-9	0-7	0-14
Mild	10-13	8-9	15-18
Moderate	14-20	10-14	19-25
Severe	21-27	15-19	26-33
Extremely Severe	28+	20+	34+

Lovibond, S.H. & Lovibond, P.F. (1995). Manual for the Depression Anxiety & Stress Scales. (2nd Ed.)Sydney: Psychology Foundation.

Outcome Questionnaire (OQ®-45.2) **Name:**__________________________ **Date:**____/_____/_______

Instructions: Looking back over the last week, including today, help us understand how you have been feeling. Read each item carefully and fill the circle completely under the category which best describes your current situation. For this questionnaire, work is defined as employment, school, housework, volunteer work, and so forth.

	Never	Rarely	Sometimes	Frequently	Almost Always
1. I get along well with others…………………………………………	O	O	O	O	O
2. I tire quickly…………………………………………………………	O	O	O	O	O
3. I feel no interest in things……………………………………………	O	O	O	O	O
4. I feel stressed at work/school………………………………………..	O	O	O	O	O
5. I blame myself for things……………………………………………	O	O	O	O	O
6. I feel irritated………………………………………………………..	O	O	O	O	O
7. I feel unhappy in my marriage/significant relationship……………	O	O	O	O	O
8. I have thoughts of ending my life……………………………….....	O	O	O	O	O
9. I feel weak………………………………………………………..…	O	O	O	O	O
10. I feel fearful………………………………………………………….	O	O	O	O	O
11. After heavy drinking, I need a drink the next morning to get……….. going. (If you do not drink, mark "never")	O	O	O	O	O
12. I find my work/school satisfying……………………………………..	O	O	O	O	O
13. I am a happy person…………………………………………………..	O	O	O	O	O
14. I work/study too much………………………………………………..	O	O	O	O	O
15. I feel worthless………………………………………………………..	O	O	O	O	O
16. I am concerned about family troubles………………………………...	O	O	O	O	O
17. I have an unfulfilling sex life………………………………………….	O	O	O	O	O
18. I feel lonely……………………………………………………………	O	O	O	O	O
19. I have frequent arguments……………………………………………	O	O	O	O	O
20. I feel loved and wanted……………………………………………….	O	O	O	O	O
21. I enjoy my spare time…………………………………………………..	O	O	O	O	O
22. I have difficulty concentrating………………………………………..	O	O	O	O	O
23. I feel hopeless about the future…………………………………….…	O	O	O	O	O
24. I like myself…………………………………………………………...	O	O	O	O	O
25. Disturbing thoughts come into [illegible] mind [illegible] cannot get rid of……..	O	O	O	O	O
26. I feel annoyed by people [illegible] [illegible] my drinking (or drug use)…... (If not applicable, [illegible] [illegible]	O	O	O	O	O
27. I have an [illegible] [illegible]……………………………………………	O	O	O	O	O
28. I am [illegible] working/studying [illegible] [illegible] as I used to………………………	O	O	O	O	O
29. My [illegible] [illegible] too [illegible]…………………………………………..	O	O	O	O	O
30. I have trouble getting [illegible] with friends and close acquaintances….	O	O	O	O	O
31. I am satisfied [illegible] [illegible] life……………………………………………	O	O	O	O	O
32. I have trouble at work/school because of drinking or drug use……… (If not applicable, mark "never")	O	O	O	O	O
33. I feel that something bad is going to happen………………………....	O	O	O	O	O
34. I have sore muscles……………………………………………………	O	O	O	O	O
35. I feel afraid of open spaces, of driving, or being on buses,………….. subways, and so forth.	O	O	O	O	O
36. I feel nervous………………………………………………………….	O	O	O	O	O
37. I feel my love relationships are full and complete……………………	O	O	O	O	O
38. I feel that I am not doing well at work/school………………………..	O	O	O	O	O
39. I have too many disagreements at work/school………………………	O	O	O	O	O
40. I feel something is wrong with my mind……………………………..	O	O	O	O	O
41. I have trouble falling asleep or staying asleep………………………..	O	O	O	O	O
42. I feel blue……………………………………………………………..	O	O	O	O	O
43. I am satisfied with my relationships with others……………………..	O	O	O	O	O
44. I feel angry enough at work/school to do something I might regret….	O	O	O	O	O
45. I have headaches………………………………………………………	O	O	O	O	O

Developed by
Michael J. Lambert, Ph.D.
and
Gary M. Burlingame, Ph.D.

© Copyright 1996 American Professional Credentialing Services LLC.
All Rights Reserved.
License Required For All Uses

For More Information Contact:

OQ Measures, LLC
P.O. Box 521047
Salt Lake City, UT 84152

Toll-Free USA:
1-888-MH-SCORE
(1-888-647-2673)

Phone: (801) 649-4392
Fax: (801) 747-6900

Email:
INFO@OQMEASURES.COM

Website:
WWW.OQMEASURES.COM

Sample

uMARS

Instructions for use:

Raters should:

1. Use the app and trial it thoroughly for at least 10 minutes;
2. Determine how easy it is to use, how well it functions and does it do what it purports to do;
3. Review app settings, developer information, external links, security features, etc.

Scoring

A: Engagement Mean Score = ________________

B: Functionality Mean Score = ________________

C: Aesthetics Mean Score = _________________

D: Information Mean Score* = ________________

* Exclude questions rated as "N/A" from the mean score calculation.

App quality mean score ___________________= A + B + C + D / 4

The *App subjective quality* scale can be reported as individual items or as a mean score, depending on the aims of the research.

The *Perceived impact* items can be adjusted and used to obtain information on the perceived impact of the app on the user's knowledge, attitudes and intentions related to the target health behaviour.

Mobile Application Rating Scale: user version (uMARS)

App Name: ____________________

Circle the number that most accurately represents the quality of the app you are rating. All items are rated on a 5-point scale from “1.Inadequate” to “5.Excellent”. Select N/A if the app component is irrelevant.

App Quality Ratings

SECTION A

Engagement – fun, interesting, customisable, interactive, has prompts (e.g. sends alerts, messages, reminders, feedback, enables sharing)

1. **Entertainment: Is the app fun/entertaining to use? Does it have components that make it more fun than other similar apps?**

   1 Dull, not fun or entertaining at all
   2 Mostly boring
   3 OK, fun enough to entertain user for a brief time (< 5 minutes)
   4 Moderately fun and entertaining, would entertain user for some time (5-10 minutes total)
   5 Highly entertaining and fun, would stimulate repeat use

2. **Interest: Is the app interesting to use? Does it present its information in an interesting way compared to other similar apps?**

   1 Not interesting at all
   2 Mostly uninteresting
   3 OK, neither interesting nor uninteresting; would engage user for a brief time (< 5 minutes)
   4 Moderately interesting; would engage user for some time (5-10 minutes total)
   5 Very interesting, would engage user in repeat use

3. **Customisation: Does it allow you to customise the settings and preferences that you would like to (e.g. sound, content and notifications)?**

   1 Does not allow any customisation or requires setting to be input every time
   2 Allows little customisation and that limits app’s functions
   3 Basic customisation to function adequately
   4 Allows numerous options for customisation
   5 Allows complete tailoring the user’s characteristics/preferences, remembers all settings

4. **Interactivity: Does it allow user input, provide feedback, contain prompts (reminders, sharing options, notifications, etc.)?**

   1 No interactive features and/or no response to user input
   2 Some, but not enough interactive features which limits app’s functions
   3 Basic interactive features to function adequately
   4 Offers a variety of interactive features, feedback and user input options
   5 Very high level of responsiveness through interactive features, feedback and user input options

[redacted] [redacted]

5. **Target group: Is the app content (visuals, language, design) appropriate for the target audience?**
   1 Completely inappropriate, unclear or confusing
   2 Mostly inappropriate, unclear or confusing
   3 Acceptable but not specifically designed for the target audience. May be inappropriate/ unclear/confusing at times
   4 Designed for the target audience, with minor issues
   5 Designed specifically for the target audience, no issues found

SECTION B

Functionality – app functioning, easy to learn, navigation, flow logic, and gestural design of app

6. **Performance: How accurately/fast do the app features (functions) and components (buttons/menus) work?**
   1 App is broken; no/insufficient/inaccurate response (e.g. crashes/bugs/broken features, etc.)
   2 Some functions work, but lagging or contains major technical problems
   3 App works overall. Some technical problems need fixing, or is slow at times
   4 Mostly functional with minor/negligible problems
   5 Perfect/timely response; no technical bugs found, or contains a 'loading time left' indicator (if relevant)

7. **Ease of use: How easy is it to learn how to use the app; how clear are the menu labels, icons and instructions?**
   1 No/limited instructions; menu labels, icons are confusing; complicated
   2 Takes a lot of time or effort
   3 Takes some time or effort
   4 Easy to learn (or has clear instructions)
   5 Able to use app immediately; intuitive; simple (no instructions needed)

8. **Navigation: Does moving between screens make sense; Does app have all necessary links between screens?**
   1 No logical connection between screens at all /navigation is difficult
   2 Understandable after a lot of time/effort
   3 Understandable after some time/effort
   4 Easy to understand/navigate
   5 Perfectly logical, easy, clear and intuitive screen flow throughout, and/or has shortcuts

9. **Gestural design: Do taps/swipes/pinches/scrolls make sense? Are they consistent across all components/screens?**
   1 Completely inconsistent/confusing
   2 Often inconsistent/confusing
   3 OK with some inconsistencies/confusing elements
   4 Mostly consistent/intuitive with negligible problems
   5 Perfectly consistent and intuitive

SECTION C

Aesthetics – graphic design, overall visual appeal, colour scheme, and stylistic consistency

10. Layout: Is arrangement and size of buttons, icons, menus and content on the screen appropriate?

1 Very bad design, cluttered, some options impossible to select, locate, see or read
2 Bad design, random, unclear, some options difficult to select/locate/see/read
3 Satisfactory, few problems with selecting/locating/seeing/reading items
4 Mostly clear, able to select/locate/see/read items
5 Professional, simple, clear, orderly, logically organised

11. Graphics: How high is the quality/resolution of graphics used for buttons, icons, menus and content?

1 Graphics appear amateur, very poor visual design - disproportionate, stylistically inconsistent
2 Low quality/low resolution graphics; low quality visual design – disproportionate
3 Moderate quality graphics and visual design (generally consistent in style)
4 High quality/resolution graphics and visual design – mostly proportionate, consistent in style
5 Very high quality/resolution graphics and visual design - proportionate, consistent in style throughout

12. Visual appeal: How good does the app look?

1 Ugly, unpleasant to look at, poorly designed, clashing, mismatched colours
2 Bad – poorly designed, bad use of colour, visually boring
3 OK – average, neither pleasant, nor unpleasant
4 Pleasant – seamless graphics – consistent and professionally designed
5 Beautiful – very attractive, memorable, stands out; use of colour enhances app features/menus

SECTION D

Information – Contains high quality information (e.g. text, feedback, measures, references) from a credible source

13. Quality of information: Is app content correct, well written, and relevant to the goal/topic of the app?

N/A There is no information within the app
1 Irrelevant/inappropriate/incoherent/incorrect
2 Poor. Barely relevant/appropriate/coherent/may be incorrect
3 Moderately relevant/appropriate/coherent/and appears correct
4 Relevant/appropriate/coherent/correct
5 Highly relevant, appropriate, coherent, and correct

14. Quantity of information: Is the information within the app comprehensive but concise?

N/A There is no information within the app
1 Minimal or overwhelming
2 Insufficient or possibly overwhelming
3 OK but not comprehensive or concise
4 Offers a broad range of information, has some gaps or unnecessary detail; or has no links to more information and resources
5 Comprehensive and concise; contains links to more information and resources

[redacted] [redacted]

15. Visual information: Is visual explanation of concepts – through charts/graphs/images/videos, etc. – clear, logical, correct?

N/A There is no visual information within the app (e.g. it only contains audio, or text)
1 Completely unclear/confusing/wrong or necessary but missing
2 Mostly unclear/confusing/wrong
3 OK but often unclear/confusing/wrong
4 Mostly clear/logical/correct with negligible issues
5 Perfectly clear/logical/correct

16. Credibility of source: does the information within the app seem to come from a credible source?

N/A There is no information within the app
1 Suspicious source
2 Lacks credibility
3 Not suspicious but legitimacy of source is unclear
4 Possibly comes from a legitimate source
5 Definitely comes from a legitimate/specialised source

App subjective quality

SECTION E

17. Would you recommend this app to people who might benefit from it?

1	Not at all	I would not recommend this app to anyone
2		There are very few people I would recommend this app to
3	Maybe	There are several people I would recommend this app to
4		There are many people I would recommend this app to
5	Definitely	I would recommend this app to everyone

18. How many times do you think you would use this app in the next 12 months if it was relevant to you?

1 None
2 1-2
3 3-10
4 10-50
5 >50

19. Would you pay for this app?

1 Definitely not
2
3
4
5 Definitely yes

20. What is your overall (star) rating of the app?

1	★	One of the worst apps I've used
2	★★	
3	★★★	Average
4	★★★★	
5	★★★★★	One of the best apps I've used

[redacted] [redacted]

Perceived impact

SECTION F

1. **Awareness – This app has increased my awareness of the importance of addressing the health behaviour**

Strongly disagree				Strongly Agree
1	2	3	4	5

2. **Knowledge – This app has increased my knowledge/understanding of the health behaviour**

Strongly disagree				Strongly Agree
1	2	3	4	5

3. **Attitudes – The app has changed my attitudes toward improving this health behaviour**

Strongly disagree				Strongly Agree
1	2	3	4	5

4. **Intention to change – The app has increased my intentions/motivation to address this health behaviour**

Strongly disagree				Strongly Agree
1	2	3	4	5

5. **Help seeking – This app would encourage me to seek further help to address the health behaviour (if I needed it)**

Strongly disagree				Strongly Agree
1	2	3	4	5

6. **Behaviour change – Use of this app will increase/decrease the health behaviour**

Strongly disagree				Strongly Agree
1	2	3	4	5

**Further comments about the app?**

THANK YOU!

[redacted] [redacted]
